# The Combination of Radiotherapy and Complement C3a Inhibition Potentiates Natural Killer cell Functions Against Pancreatic Cancer

**DOI:** 10.1158/2767-9764.CRC-22-0069

**Published:** 2022-07-27

**Authors:** Quaovi H. Sodji, Dhanya K. Nambiar, Vignesh Viswanathan, Rie von Eyben, Deana Colburg, Michael S. Binkley, Caiyun G. Li, Monica M. Olcina, Daniel T. Chang, Quynh-Thu Le, Amato J. Giaccia

**Affiliations:** 1 Department of Radiation Oncology, Stanford University School of Medicine, Stanford, California.; 2 Department of Pathology, Stanford University School of Medicine, Stanford, California.; 3 MRC/CRUK Oxford Institute for Radiation Oncology and Gray Laboratory, University of Oxford, Oxford, United Kingdom.

## Abstract

Pancreatic cancer is one of the deadliest cancers, against which current immunotherapy strategies are not effective. Herein, we analyzed the immune cell composition of the tumor microenvironment of pancreatic cancer samples in The Cancer Genome Atlas and found that the presence of intratumoral natural killer (NK) cells correlates with survival. Subsequent analysis also indicated that NK cell exclusion from the microenvironment is found in a high percentage of clinical pancreatic cancers and in preclinical models of pancreatic cancer. Mechanistically, NK cell exclusion is regulated in part by complement C3a and its receptor signaling. Inhibition of the C3a receptor enhances NK cell infiltration in syngeneic mouse models of pancreatic cancer resulting in tumor growth delay. However, tumor growth inhibition mediated by NK cells is not sufficient alone for complete tumor regression, but is potentiated when combined with radiotherapy. Our findings indicate that although C3a inhibition is a promising approach to enhance NK cell–based immunotherapy against pancreatic cancer, its combination with radiotherapy holds greater therapeutic benefit.

**Significance:**

Immunotherapeutic agents are not effective against pancreatic cancer. We show that the inhibition of complement C3a enhances NK cell infiltration in preclinical models of pancreatic cancer, resulting in tumor growth delay. This effect is further potentiated by radiotherapy, thereby leading to significant antitumor activity compared with either therapy alone.

## Introduction

In 2021, there were over 60,000 new cases and 48,000 deaths due to pancreatic cancer in the United States, making this cancer the third leading cause of cancer death (1). Mortality from pancreatic cancer has been on a steady rise and is projected to become the second leading cause of cancer deaths in the United States by 2030, only behind lung cancer (2). Pancreatic cancer has also become a worldwide problem as there were 495,000 new diagnoses of pancreatic cancer and 466,000 deaths due to this cancer in 2020 (3). Despite recent advances in systemic therapies, the 5-year relative survival of pancreatic cancer remains low at 10.8%, thus making this cancer fatal in the majority of patients (1). In addition to its significant mortality, the estimated global disability-adjusted life years of pancreatic cancer has been trending up and is currently estimated at 9.1 million years (4). Furthermore, with the increase in the incidence and mortality of pancreatic cancer observed worldwide, its societal burden will continue to rise (5). As such, a paradigm shift is needed in the treatment of pancreatic cancer.

Immune checkpoint inhibitors (ICI) have revolutionized cancer management. Inhibitors for CTLA-4 or PD1/PDL1, critical immunomodulators which target T cells, are used either as monotherapy or in combination therapy to treat over 50 different cancer types and are currently being evaluated in more than 3,400 clinical trials (6, 7). However, despite promising results against many solid tumors, ICIs have not been effective against pancreatic cancer for a number of reasons that include a low mutational burden, a microenvironment characterized by significant desmoplasia and hypoxia, and the presence of immunosuppressive cells such as tumor-associated macrophages, regulatory T cells, and myeloid-derived suppressor cells (MDSC; refs. 8, 9). Other immunotherapy strategies such as vaccine therapy and adoptive cell transfer have also yielded disappointing results, highlighting the need for novel immunotherapy approaches in pancreatic cancer (10).

Natural killer (NK) cells are cytotoxic lymphocytes of the innate immune system with rapid cytolytic activity against malignant and virus-infected cells without prior sensitization (11). NK cells still retain their cytotoxic activity against malignant cells that downregulate the expression of MHC class I (MHC-I) molecules to evade the immune system surveillance (12). The cytotoxic activity of NK cells against malignant cells is contingent on their infiltration into the tumor microenvironment (TME) and there is increasing evidence that high intratumoral density of NK cells correlates with improved survival in various cancers including colorectal, gastric, lung, renal, esophageal, breast, and head and neck cancers (13–19).

Our previous study showed that complement C3a, a key peptide of the innate immune system involved in a host defense against pathogens, is also involved in the exclusion of NK cells from the TME of breast and colon cancers (20, 21). The binding of C3a to its receptor, C3aR on NK cells, leads to the interaction of the C3aR with the integrin lymphocyte function–associated antigen 1 (LFA-1), resulting in a conformational change in LFA-1 and a decreased infiltration of NK cells in the TME. Furthermore, disruption of the C3a/C3aR axis restores NK cells infiltration (21).

Herein, we provide evidence that NK cell infiltration in the TME of patients with pancreatic cancer correlates with survival. We also investigate the role of C3a secreted by pancreatic cancer cell lines on the migration of NK cells into the TME of pancreatic cancers. We determined the effect of inhibiting C3aR with the small-molecule antagonist SB290157 in a syngeneic mouse model of pancreatic cancer and its enhancement of NK cell infiltration in the TME and on tumor growth delay. Because altering NK cell migration alone did not result in tumor eradication, we also determined whether C3aR inhibition is potentiated by radiotherapy in preclinical pancreatic cancer mouse models. Our findings suggest that targeting complement C3a increases NK infiltration into experimental pancreatic tumors and this novel immunotherapeutic approach against pancreatic cancer can be potentiated by radiotherapy.

## Materials and Methods

### Patients and Samples

Written informed consents were obtained from all patients involved in this study which was conducted in accordance with the Declaration of Helsinki and approved by the Stanford University Institutional Review Board. Tumor samples were obtained from patients with pancreatic cancer following surgical resection. The pancreatic cancer tissue microarrays (TMA) were constructed from archived formalin-fixed paraffin-embedded tumor samples. Three cores of 2 mm sampled from various areas of the tumor were used for each patient to construct the TMAs.

### Cell Culture

The human pancreatic cancer cell lines PANC-1, MIAPaCa-2 (kindly donated by Dr. Laura Attardi, Stanford University, Stanford, CA in July 2019) and murine pancreatic cancer cell line Pan02 (kindly donated by Dr. Edgar Engleman, Stanford University, Stanford, CA in October 2019) were grown in DMEM supplemented with 10% FBS, 2% glutamine, 1% pen/strep at 37°C and 5% CO_2_. Cell line authentication was not performed after the reception of the donated cell lines. These cell lines were often tested to rule out *Mycoplasma* contamination using a *Mycoplasma* detection assay (Lonza, #LT07-703).

### Mouse Models and Treatments

All aspects of animal experiments, handling, and procedures were approved by the Institutional Animal Care and Use Committee at Stanford University (Stanford, CA) in accordance to all regulations. The mice were housed in animal facilities at Stanford University (Stanford, CA) and fed *ad libitum*.—Six to 8 weeks old C57BL/6J and nude (*Foxn1^nu^/Foxn1^nu^)* mice were purchased from Jackson Laboratory (JAX:000664; JAX:007850). A total of 5 million cells of PANC-1 and MIAPaCa-2 were implanted subcutaneously on the right flank of nude mice, whereas 600,000 cells of Pan02 cells were implanted subcutaneous on the right flank of C57BL/6J mice. The tumor size was measured using an electronic caliper and the volume was calculated using the formula: (*D* × *d*^2^)/2; with *D* representing the length along the major axis and *d* that along the minor axis. Mice were randomized into the various treatment arm when the tumor volume reached approximately 100 mm^3^. In the xenograft models, C3a neutralization was achieved by the intraperitoneal injection of 100 μg in PBS of human anti-C3a antibody (BioLegend, #518106) per mouse every other day for a total of five doses. A 10 mmol/L stock solution of the C3aR antagonist small molecule SB290157 (Cayman Chemical, #15783) was made in DMSO and subsequent dilutions were made in PBS for daily intraperitoneal injection. In the xenograft models, mice were administered daily SB290157 (7.5 mg/kg, 4% DMSO in PBS) or vehicle (4% DMSO in PBS) for 22 days. In the syngeneic model, mice were administered daily SB290157 (5 mg/kg, 4% DMSO in PBS) or vehicle (4% DMSO in PBS) for 14 or 22 days.

### CIBERSORTx Analysis

The digital deconvolution tool, CIBERSORTx (https://cibersortx.stanford.edu) with a validated signature matrix for distinguishing 22 human hematopoietic cell subsets in bulk tissues including tumors was applied to RNA sequencing (RNA-seq) data from patients with pancreatic cancer available from The Cancer Genome Atlas (TCGA; ref. 22). Pancreatic cancer TCGA data were downloaded from the International Cancer Genome Consortium (http: dcc.icgc.org on 07/06/2020). Among the 185 patients in the database, RNA-seq data were available for 142 patients. We subsequently excluded from our analysis patients with neuroendocrine histology, resulting in 138 analyzable patients. Kaplan–Meier survival curves based on the relative proportion of total NK cells (activated + resting NK cells) and CD8^+^ T cells were plotted with GraphPad Prism and analyzed using the log-rank test and subsequent significance was defined as a *P* < 0.05. Survival was defined as the time from diagnosis to death.

### Histopathology

Mice tumors were fixed in 10% formalin for 24 hours and stored in 70% ethanol and subsequently embedded in paraffin. Sections were stained in hematoxylin and eosin (H&E) or trichrome stains. After antigen retrieval, the sections were blocked with 2.5% normal goat serum (Vector laboratories, #S-1012) and incubated overnight at 4°C with the following antibodies for NK cell staining: Anti-NCR1 (Abcam, #ab214468, 1:200) or FITC anti-mouse NK1.1 antibody (BioLegend, #108705, 1:100). Nuclei were stained with DAPI Fluoromount-G (SouthernBiotech, #0100-20). The slides were visualized using the Leica DMi8 microscope. For the TMAs, IHC staining was performed as described previously and stained for NKp46 (R&D Systems, MAB1850, 1:100) and CD56 (Cell Marque, 156M-86, 1:100; ref. 23). CD56^+^ cells were counted on each core by an observer blinded to the clinical patient data.

### ELISA

The supernatant of PANC-1 and MIAPaCa-2 grown in phenol red–free DMEM with 10% FBS, 2% glutamine, and 1% pen/strep at 37°C and 5% CO_2_ was collected, divided in aliquot, and stored at −80°C. C3a and C5a in the supernatant were subsequently measured according to the manufacturer's protocol using a C3a ELISA kit (HycultBiotech, HK354-01) and C5a ELISA kit (HycultBiotech, HK349-01).

### NK Cell Isolation

Blood from healthy volunteer donors was obtained from the Stanford Blood Center. Peripheral blood mononuclear cells were prepared from the blood using Ficoll-Paque PLUS (GE Healthcare, #17-1440-02). NK cells were isolated using an immunomagnetic negative selection from the EasySep™ Human NK Cell Isolation Kit (STEMCELL Technologies, #17955) according to the manufacturer's protocol. Murine NK cells were isolated from murine spleens using the immunomagnetic negative selection from the EasySep™ Mouse NK Cell Isolation Kit (STEMCELL Technologies, #19855) according to the manufacturer's protocol. The isolated NK cells were maintained in RPMI, supplemented with 10% FBS, 2% glutamine, 1% pen/strep, and recombinant human IL2 (1,000 U/mL; PreproTech, #200-02) at 37°C in 5% CO_2_.

### NK Cell Cytotoxic Activity

The CytoTox 96 Non-Radioactive Cytotoxicity Assay (Promega, #G1780) was used to measure the NK cell cytotoxic activity as reported previously (24). Briefly, 10^4^ target cells in 50 μL of phenol red–free RPMI media supplemented with heat-inactivated 10% FBS, 2% glutamine was added to each well on a U-bottom–shaped 96-well plate. Seventy-two hours after their isolation, effector (NK) cells in 50 μL/well were added at various ratios of effector and target cells ranging from 2.5:1 to 20:1 and incubated for 4 hours. The lactate dehydrogenase (LDH) activity was measured in the supernatant according to the manufacturer's protocol.

The % cytotoxicity was calculated as followed:



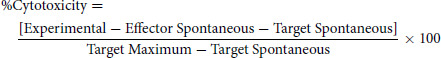



Effector Spontaneous: LDH spontaneously released by NK cells.

Target Spontaneous: LDH spontaneously released by tumor cells.

Target Maximum: Maximum LDH activity after complete lysis of all tumor cells.

### Three-Dimensional Collagen Migration Assay

Collagen matrix containing tumor cells was prepared by mixing type I bovine collagen (Advanced BioMatrix, #5005; 55%), a 7.5% solution of NaHCO_3_ (3%), sterile deionized water (16%), a 10× MEM (7%), and DMEM containing tumor cells (18%). The collagen solution containing the tumor cells was prepared and kept on ice before 400 μL was poured inside a glass culture cylinder (Automate Scientific, #Bi-070303) placed into a well on a 12-well plate. After incubation at 37°C in 5% CO_2_ for 40 minutes, few drops of DMEM were added to the solidified collagen matrix and the setup was incubated for 24 hours at 37°C in 5% CO_2_. After 24 hours, a collagen matrix containing NK cells labeled with green CMFDA dye (Invitrogen, #C2925) was prepared with type I bovine collagen (55%), a 7.5% solution of NaHCO_3_ (3%), sterile deionized water (16%), a 10× MEM (7%), and RPMI containing NK cells (18%). A total of 500 μL of the collagen containing NK cells was poured outside of the glass culture cylinder and the setup was incubated for 30 minutes at 37°C in 5% CO_2_. After the incubation, the glass culture cylinder was removed and 75 μL of RPMI was added along the wall of the well. After 24 hours incubation at 37°C in 5% CO_2_, the setup was visualized under fluorescence microscopy (Leica DMi8) to evaluate NK cell distribution. ImageJ was used to quantify NK cells within the collagen matrix containing tumor cells and reported as # of NK cells per 10^4^ μm^2^. For C3a neutralization experiment, anti C3a antibody (BioLegend, 518105) was added during the preparation of the collagen matrix containing tumor cells to a final concentration of 25 μg/mL.

### Tumor Cell Isolation

Mice were euthanized and the tumors were harvested and placed in 1 mL of cold FBS free RPMI. The tumors were cut into smaller pieces with a scalpel and 1 mL of Tumor Dissociation Buffer (Miltenyi Biotec, #130-096-730) was added and incubated at 37°C for 40 minutes with continuous shaking at 90 rpm. After the incubation, the dissociation was quenched by the addition of 3 mL of RPMI with 10% FBS and the samples were filtered through a 70 μm filter. After centrifugation at 1,500 rpm for 5 minutes, the supernatant was discarded and the pellet was resuspended in PBS.

### Immune Cell Profiling

Tumors were harvested and resuspended into single-cell solution in PBS as described above. Murine spleens were mashed through a 70 μm filter into a ACK lysing buffer (Gibco, #A1049201) and incubated for 3 minutes. A total of 10 mL of PBS was added after the RBC lysis and the suspension was centrifuged at 1,500 rpm for 5 minutes. The supernatant was discarded and the pellet was resuspended in PBS. After Fc blocking (BioLegend, 101319; 1:100) and Live/Dead staining with zombie NIR (BioLegend, #423105; 1:1,000) was performed during a 10-minute incubation at 4°C, the samples were washed with PBS. Conjugated antibodies including CD45-BV711 (BioLegend, #103147; 1:200), CD4-PE (BioLegend, #100407; 1:100), CD8-Pe-Cy7 (BioLegend, #100721; 1:100), NK1.1-FITC (BioLegend, #108705; 1:100), B220-Pe-Cy5.5 (BioLegend, #103209; 1:100), CD11b-APC (BioLegend, #101211; 1:200), F4/80-BV785 (BioLegend, #123141; 1:200), GR1-Pe Tx red (BD Biosciences, # 562710; 1:200) were incubated with each sample at 4°C in the dark for 20 minutes and washed with FACS buffer (2% FBS in PBS). The samples were analyzed using BD LSRFortessa X-20 flow cytometer. Collected flow cytometry data were analyzed using FlowJo software.

### NK Cell or CD8^+^ T-Cell Depletion *in vivo*

C57BL/6 mice bearing Pan02 tumor were randomized after tumor size reached approximately 100 mm^3^. The mice were injected intraperitoneally 100 μg of NK cell depletion antibody (BioLegend, #108702) or CD8^+^ T-cell depletion antibody (BioLegend, #155012) in PBS, starting on day 0 and every 4 days. Mouse IgG1 (BioLegend, #400166) was used as antibody isotype control. Mice treated with the aforementioned antibodies and isotype control were treated with daily with the C3aR antagonist SB290157 (5 mg/kg) for 14 days. A control arm was treated with vehicle (4% DMSO in PBS).

### Mice Irradiation

The mice were anesthetized with isoflurane and the small animal irradiator (Precision X-ray Inc, X-rad SmART) was used to acquire CT images which were subsequently used to design a radiation treatment plan. A single dose of 5 Gy in 1 fraction was delivered with AP/PA beam arrangements using a 10-mm circular collimator.

### Statistical Analysis

To analyze the CIBERSORTx output, a recursive partitioning analysis was performed to dichotomize patients into high versus low CD8^+^ T cells or NK cells, using overall survival as an endpoint. A split was performed if there were at least 46 patients (one-third of the entire cohort) in each subgroup and the *P* value was statistically significant. In addition, subgroups without significant difference in survival outcome were combined. Patient and clinical characteristics were summarized using means, SDs, medians, ranges, and proportions as appropriate. NK cell infiltration in the TME, cytotoxic activity, number of days to reaching 300 and 500 mm^3^ in xenograft mouse models were analyzed using *t* test when comparing two groups. The number of days to reaching 300 mm^3^ in the syngeneic models were analyzed using ANOVA models when comparing more than two groups. The groups were compared pairwise in a *post hoc* analysis with a Tukey adjustment for multiple comparisons. Time-to-event outcomes were summarized using Kaplan–Meier curves and groups were compared using log-rank tests. All tests were two sided with an alpha level of 0.05. All analyses were performed in SAS v9.4 (SAS Institute Inc) and all graphs were generated in Prism v9.3 (GraphPad Software).

### Data Availability Statement

The data generated in this study are available within the article and its Supplementary Data.

## Results

### High NK Cell Density in the TME of Pancreatic Cancer Correlates with Improved Survival

**FIGURE 1:**
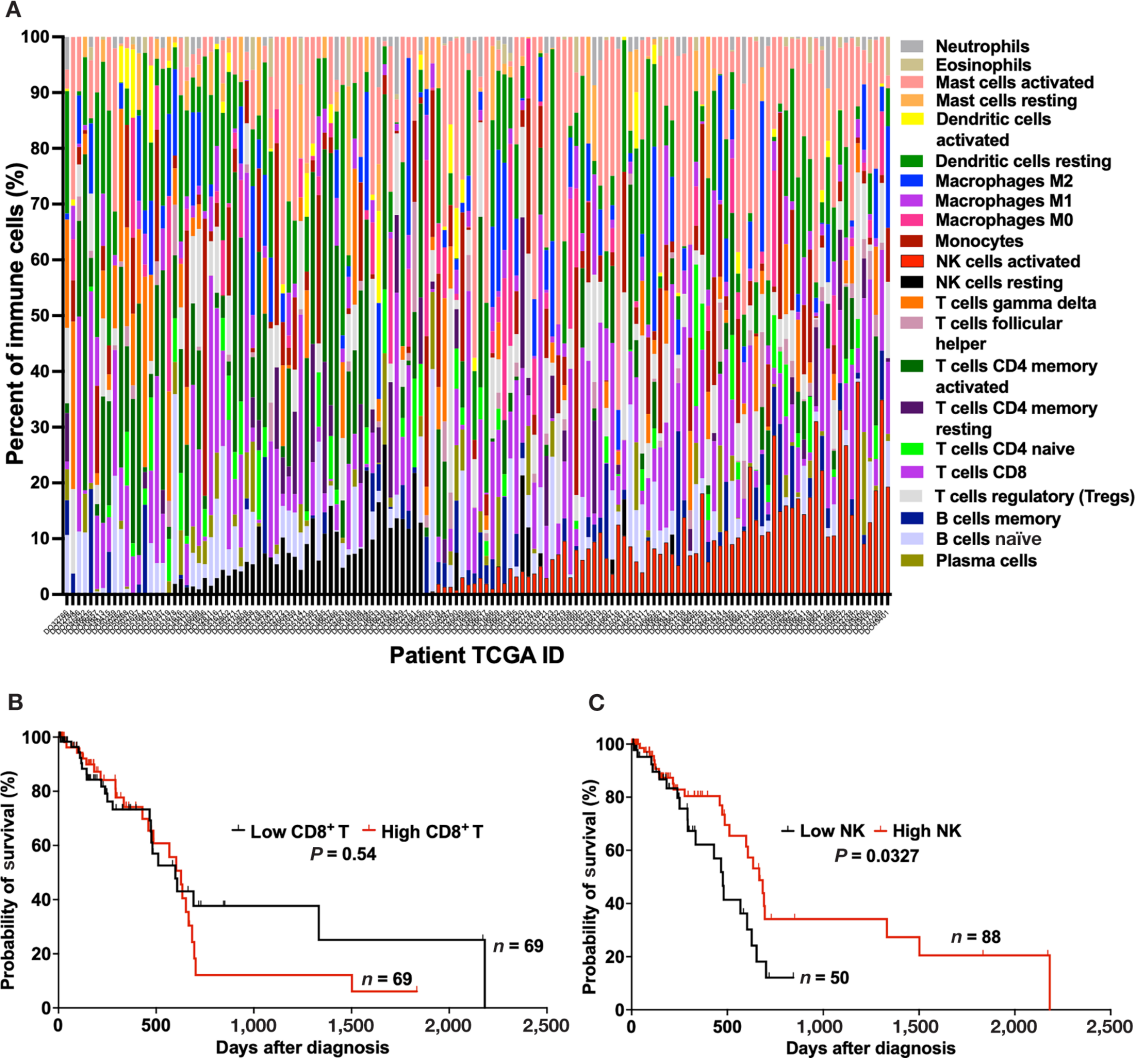
Kaplan–Meier curves of overall survival based on CD8^+^ T cells and NK cells in the TME of 138 patients with pancreatic cancer: CIBERSORTx analysis of TCGA database. **A,** Proportion of various immune cells type in the TME of 138 patients with pancreatic cancer based on the CIBERSORTx analysis of TCGA. Each column represents a patient with pancreatic cancer and each color is indicative of an immune cell type. **B,** The density of CD8^+^ T cells in the TME of patients with pancreatic cancer in TCGA database does not correlate with survival. Median survival for patients with high (>8.52%, *n* = 69; red) versus low (<8.52%, *n* = 69; black) CD8^+^ T cells: 627 and 598 days, respectively (*P* = 0.54, log-rank test). **C,** Pancreatic cancer patients in TCGA database with high proportion of NK cells in the TME have better survival. Median survival for patients with high (>5%, *n* = 88; red) versus low (<5%, *n* = 50; black) NK cells: 666 and 476 days, respectively (*P* = 0.0327, log-rank test).

To evaluate whether the density of NK cells in the TME of pancreatic cancer correlates with patients’ survival, we first determined the relative proportion of 22 immune cell types in the TME using CIBERSORTx (cell-type identification by estimating relative subsets of RNA transcripts). We applied CIBERSORTx to RNA-seq data of pancreatic cancers from 138 patients in TCGA database (22). The relative proportion of the 22 immune cell types in the TME of pancreatic patients is shown in Fig. 1A and Supplementary Table S1. For total NK cells (Supplementary Fig. S1), partitioning analysis identified 5% as the optimal point to split patients into high (total NK cells >5%) versus low NK cells (total NK cells <5%). This value corresponds to the 70th percentile in a previous study which evaluated the total NK cells in the normal pancreas (25). For CD8^+^ T cells, no significant cut-off point to dichotomize the patients was identified, thus the median was reported. We subsequently constructed Kaplan–Meier curves based on the relative proportion of CD8^+^ T cells and total NK cells, the key cytotoxic effector cells present in the TME. The relative proportion of CD8^+^ T cells in the TME does not correlate with survival in patients with pancreatic cancer (Fig. 1B). Patients with high CD8^+^ T cells (CD8^+^ T cells >8.52%) in their TME had a median survival of 627 days compared with 598 days for those with low CD8^+^ T cells (CD8^+^ T cells <8.52%; *P* = 0.54, log-rank test). On the other hand, the relative proportion of total NK cells in the TME of patients with pancreatic cancer does correlate with survival, as the cohorts with high (total NK cells >5%) and low NK cells (total NK cells <5%) had median survival of 666 and 476 days, respectively (*P* = 0.0327, log-rank test; Fig. 1C). These data suggest that similar to other cancers including colorectal, gastric, lung, renal, esophageal, breast, and head and neck cancers, NK cell infiltration in the TME of pancreatic cancer correlates with patients’ overall survival (13–15, 17, 18, 26, 27).

### NK Cells are Excluded from the TME of Most Patients with Pancreatic Cancer

**FIGURE 2:**
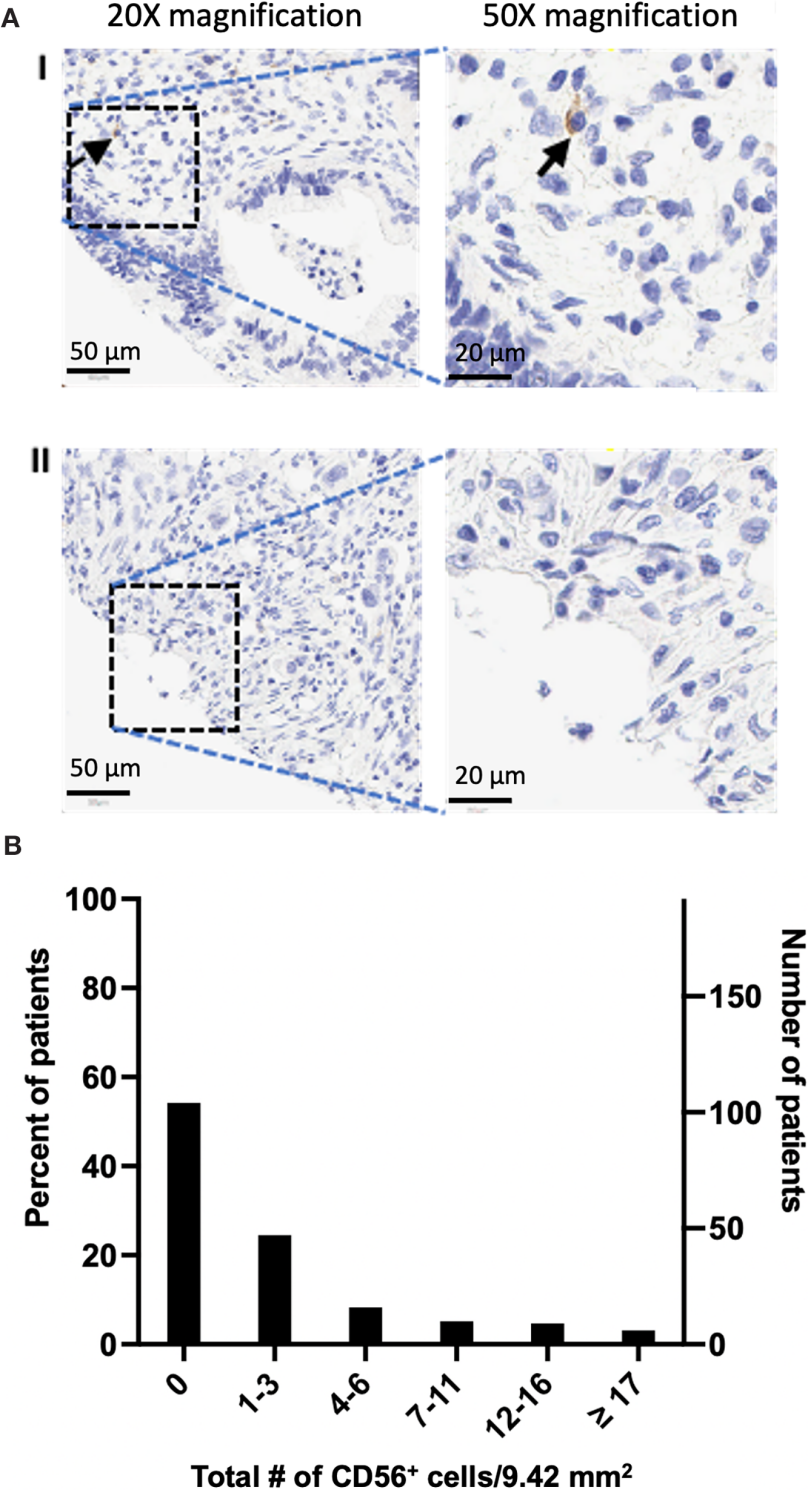
CD56^+^ cells are excluded from the TME of most patients with pancreatic cancer. **A,** Sample IHC staining of CD56^+^ cells (black arrow) on pancreatic cancer TMA. I, Representative image of pancreatic cancer with intratumoral CD56^+^ cells. 20× magnification, scale bar: 50 μm; 50× magnification, scale bar: 20 μm. II, Representative image of pancreatic cancer without CD56^+^ cells. 20× magnification, scale bar: 50 μm; 50× magnification, scale bar: 20 μm. **B,** Distribution of CD56^+^ cells in the TME of a cohort of 192 patients with pancreatic cancer in our institutional database. IHC staining for CD56^+^ cell on a TMA (three cores per patient) revealed that the TME in most patients (104 patients, 54.2%) is devoid of CD56^+^ cells. *X*-axis represents the total NK cells in three cores of TMA (9.42 mm^2^) per patient. Right *Y*-axis: Number of patients; Left *Y*-axis: percent of total patients. CD56^+^ cells were counted on each TMA core by an observer blinded to patients’ clinical outcome and data.

We next determined the presence of NK cells in the TME of a cohort of patients with pancreatic cancer in our institutional database. We constructed a TMA (three cores/patient) using samples from 192 patients with pancreatic cancer (Table 1) and performed IHC staining, for CD56 and NKp46 (Supplementary Fig. S2), NK cell surface markers (28). Because of the naturally occurring variation in NKp46 expression in human NK cells and because NKp46 expression is more dynamic than CD56, we selected CD56 as the NK cell marker to be used for our analysis (19). Figure 2A shows representative CD56^+^ cell staining on a TMA core at 20× and 50× magnifications. The total number of CD56^+^ cells present in all the cores (total surface area of 9.42 mm^2^/patient) were counted and the distribution of the CD56^+^ cells in the TME was plotted (Fig. 2B). In our cohort of 192 patients at Stanford Hospital, 104 (54.2%) had no CD56^+^ cell on their TMA cores; 47 patients (24.5%) had only—one to three CD56^+^ cells, 16 patients (8.3%) had —four to six CD56^+^ cells, 10 patients (5.2%) had —seven to 11 CD56^+^ cells, 9 patients (4.7%) had 12–16 CD56^+^ cells, and 6 patients (3.1%) had 17 or more CD56^+^ cells.

**TABLE 1:** Clinical characteristics of the 192 patients from institutional database included on the pancreatic cancer TMAs stained for CD56^+^ cells

Characteristic	*n* (%)
**Sex**	
Male	111 (58)
Female	81 (42)
**Age (years)**	
Median (range)	69 (37–86)
**T-stage** ^a^	
T1-T2	49 (26)
T3-T4	112 (58)
Unknown	31 (16)
**N-stage** ^a^	
N0	65 (34)
N1	63 (33)
N2	62 (32)
Unknown	2 (1)
**Grade**	
Poor	33 (17.2)
Poor to moderate	31 (16.1)
Moderate	103 (53.6)
Moderate to well	5 (2.6)
Well	15 (7.8)
Unknown	6 (3.1)
**Surgery**	
Whipple	126 (66)
Other^b^	66 (34)
**Margins status**	
Negative	129 (67)
Positive	63 (33)
**Perineural invasion**	
Yes	141 (73)
No	23 (12)
Unknown	28 (15)

^a^AJCC TNM Staging of Pancreatic Cancer (8th edition).

^b^Pancreaticoduodenectomy, distal pancreatectomy, total pancreatectomy, subtotal pancreatectomy, unknown.

### Pancreatic Cancer Cell Lines Secrete C3a and High-level of C3a Secretion Inhibits *in vitro* NK cell Migration

**FIGURE 3:**
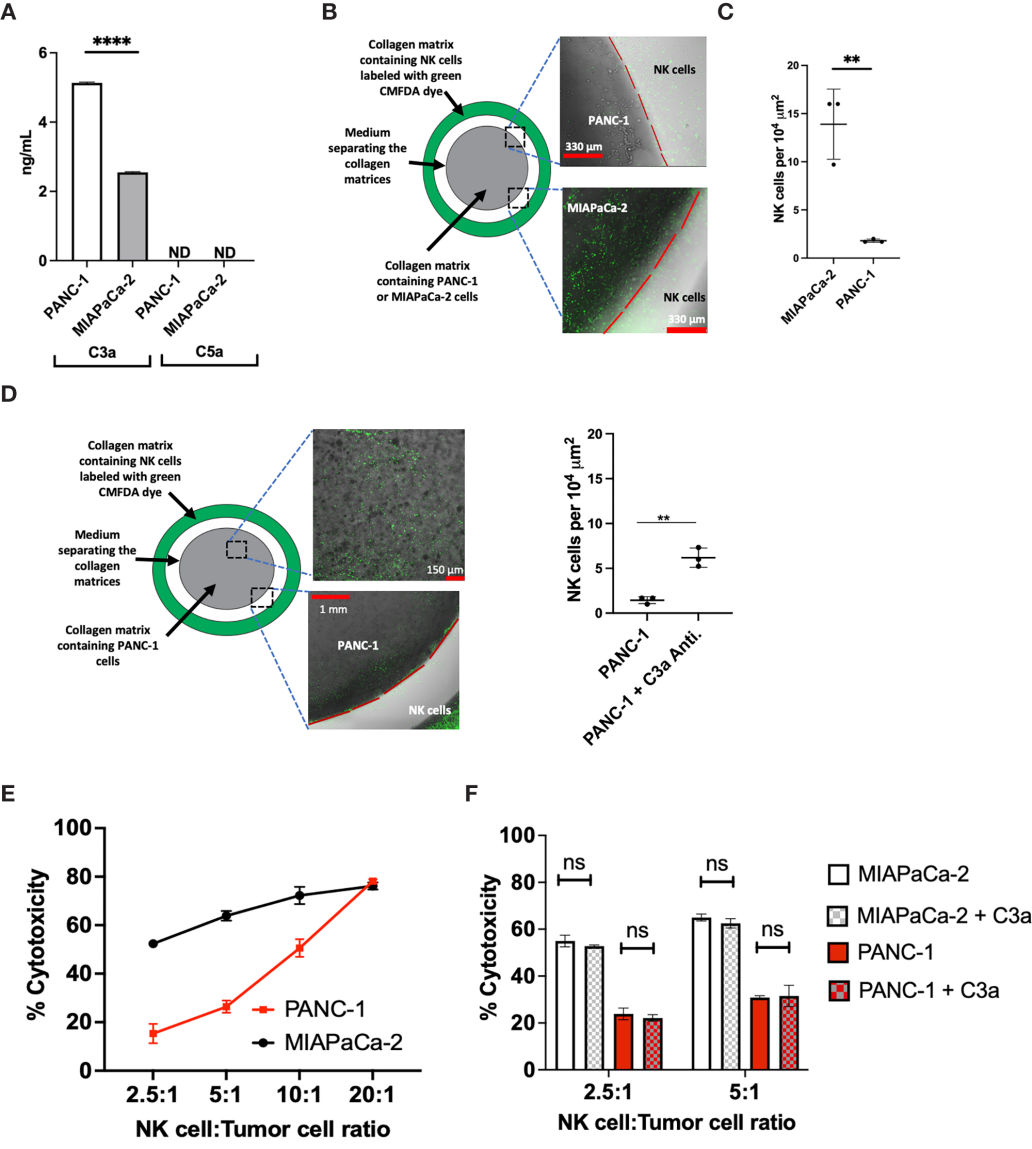
The complement C3a is secreted by human pancreatic cancer cell lines and higher concentration of C3a is associated with NK cell exclusion from the TME but C3a does not inhibit the cytotoxic activity of NK cells. **A,** C3a is secreted in the supernatant of PANC-1 and MIAPaCa-2 (****, *P* < 0.0001, *n* = 3, triplicate measurements from two independents experiments); however, C5a is not detected. C3a and C5a measured by ELISA. ND: Not detected. **B,** Experimental setup to evaluate NK cell (green CMFDA dye labeled) infiltration into a 3D collagen matrix containing human pancreatic cancer cells. The gap (white) between the collagen matrices containing tumor and NK cells is filled with media. **C,** NK cells infiltrate a 3D collagen matrix containing MIAPaCa-2, whereas NK cells do not infiltrate a 3D collagen matrix containing PANC-1 cells (**, *P* = 0.0001, *n* = 3, triplicate measurements from three independents experiments). **D,** Neutralization of C3a with human C3a antibody enhances NK cell infiltration into a 3D collagen matrix containing PANC-1 cells (**, *P* = 0.0045, *n* = 3, triplicate measurements from three independents experiments). **E,** Isolated NK cells from human donor possess cytotoxic activity against human pancreatic cancer cells. The cytotoxic activity of NK cells is measured using the LDH release assay at different NK:Tumor cells ratio (*n* = 3, three independents experiments). **F,** The complement C3a does not inhibit the cytotoxic activity of NK cells. At the NK:Tumor ratio of 2.5:1 and 5:1, addition of C3a (5 μg/mL) has no significant inhibitory effect on the cytotoxic activity of NK cells against both PANC-1 and MIAPaCa-2 cell lines. (*n* = 3, triplicate measurements from two independents experiments). ns: not significant. Statistical significance determined using *t* test.

To evaluate the production and secretion of the complement C3a by human pancreatic-derived cancer cells, we measured C3a in the supernatant of PANC-1 and MIAPaCa-2 by ELISA. We found that C3a is present in the supernatant of PANC-1 and MIAPaCa-2 at concentrations of 5.13 ± 0.02 ng/mL and 2.55 ± 0.01 ng/mL, respectively (Fig. 3A). However, C5a, another key anaphylatoxin of the complement system is not detected in the supernatant of these pancreatic cancer cell lines (Fig. 3A).

To determine the effect of the secreted C3a on the migration of NK cells, an *in vitro* three-dimensional (3D) collagen model of tumor cell culture surrounded by fluorescently labeled NK cells was developed. The migration of NK cells into collagen containing pancreatic tumor cells after 24 hours was evaluated using fluorescence microscopy. We found that NK cells isolated from human donors migrate into the collagen matrix containing MIAPaCa-2 cells, which secrete low C3a level, but not PANC-1 cells, which secrete higher level of C3a (Fig 3B and C). The neutralization of C3a with anti-C3a antibody restores NK cell migration into the collagen matrix containing PANC-1 cells (Fig. 3D).

### NK Cells Isolated from Healthy Human Donors Possess Cytotoxic Activity Against Pancreatic Cancer Cells and Such Cytotoxic Activity is not Inhibited by C3a

NK cell cytotoxicity against pancreatic cancer cells is crucial for NK cell–based immunotherapy for pancreatic cancer treatment. As such, we evaluated the cytotoxic activity of NK cells isolated from healthy human donors against PANC-1 and MIAPaCa-2 using the LDH release assay. Against both cell lines, NK cells display a potent cytotoxicity at ratio of NK cells to tumor cells ranging from 2.5:1 to 20:1 (Fig. 3E).

Although a previous report suggested that C3a could inhibit the cytotoxic activity of NK cells, in this study, the presence of C3a, up to concentration of 5 μg/mL did not impact the cytotoxic activity of NK cells against both PANC-1 and MIAPaCa-2 cell lines (Fig. 3F; ref. 29).

### Inhibition of the C3a/C3aR Axis in Xenograft Mouse Models Increases NK Cell Infiltration in the TME and Leads to Tumor Growth Delay

**FIGURE 4:**
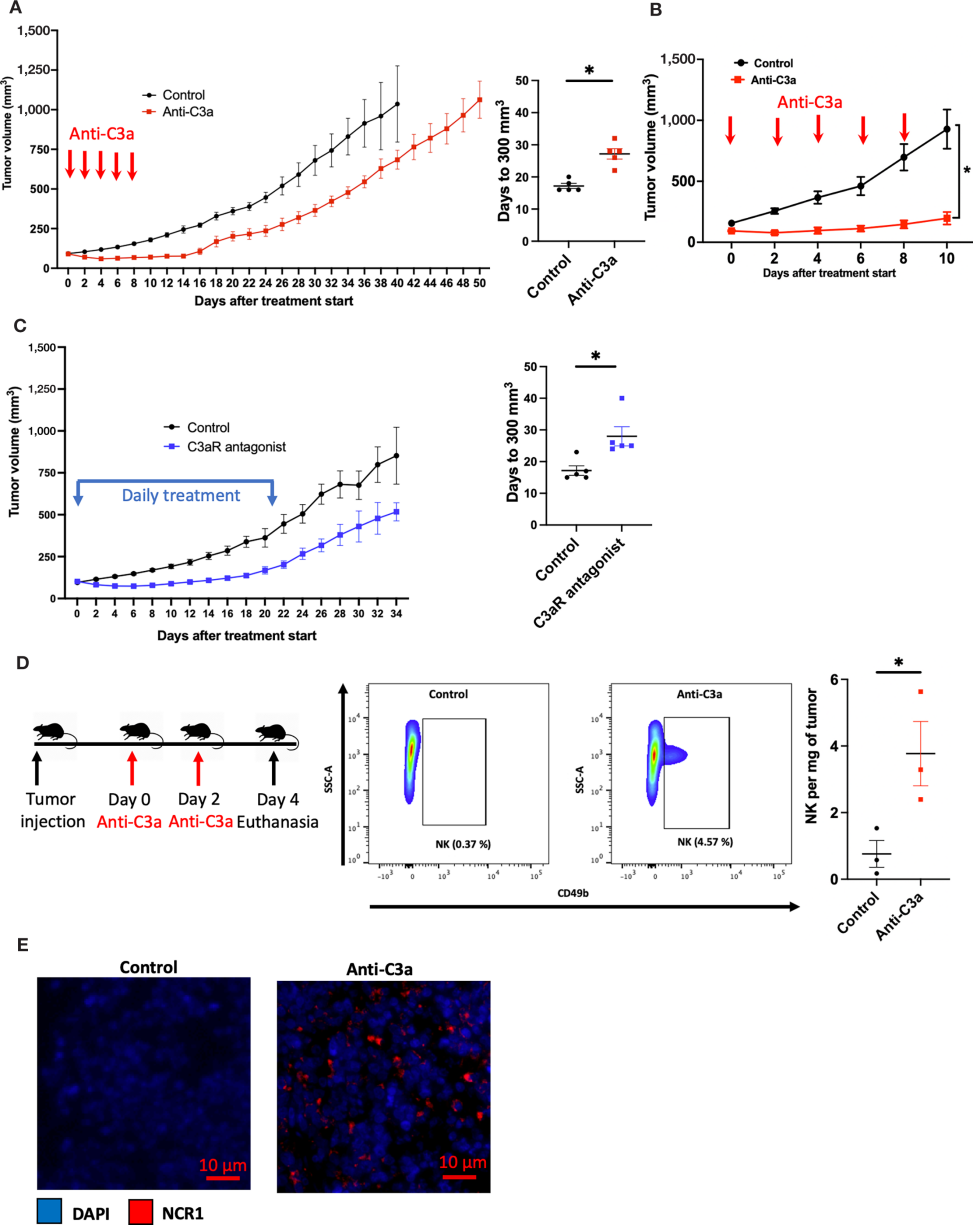
Inhibition of the C3a/C3aR axis results in tumor growth delay due to an increase of NK cells in the TME of pancreatic cancer xenograft mouse models. Tumor cells were injected subcutaneously in nude mice (Athymic nude *Foxn1^nu^*) and tumors were allowed to reach approximately 100 mm^3^ and were randomized into each treatment arm. Tumor size was monitored until experimental endpoint (tumor ulceration or volume >1,500 mm^3^). **A,** Mice bearing PANC-1 tumors were treated with Intraperitoneal injection of PBS (control, *n* = 5) or C3a antibody (Anti-C3a group, *n* = 5) every other day. Mean time to reaching 300 mm^3^ is 17.2 days in the control arm compared with 27.2 days in the Anti-C3a arm (*, *P* = 0.0006). **B,** Mice bearing MIAPaCa-2 tumor were treated with intraperitoneal injection of PBS (control, *n* = 5) or C3a antibody (anti-C3a group, *n* = 5) every other day. Anti-C3a treatment results in tumor growth delay (*, *P* < 0.0001). **C,** Mice bearing PANC-1 tumor were treated with either daily intraperitoneal injection of vehicle (4% DMSO in PBS) (control group, *n* = 5) or with daily intraperitoneal injection of the small-molecule C3aR antagonist SB290157 (C3aR antagonist group, *n* = 5, 7.5 mg/kg) for 22 days. The mean time to reaching 300 mm^3^ in the control group was 17 days compared with 28 days in the C3aR antagonist group (*, *P* < 0.0125). **D,** Neutralization of C3a enhances NK cell infiltration in the TME of PANC-1 xenograft mouse model on flow cytometry analysis. After PANC-1 tumor cells implanted subcutaneously reached a volume of 150 mm^3^, treatment was started with either intraperitoneal injection of PBS (control group, *n* = 3) or with intraperitoneal injection of C3a antibody (anti-C3a group, *n* = 3). The mice were euthanized after two treatments on day 4. FACS analysis revealed an increase in the proportion of NK cells (CD49b^+^) per tumor weight (mg) (* *P* = 0.04). **E,** IF staining of MIAPaCa-2 xenograft model revealed an increase of NK cells (NCR1, red) in the anti-C3a group compared with the control group. Statistical significance determined using *t* test otherwise specified.

We subsequently sought to determine *in vivo* the effect of inhibiting the C3a/C3aR axis in xenograft mouse models of pancreatic cancer. Athymic mice, lacking functional T cells but having functional NK cells and a complement system, were subcutaneously implanted with PANC-1 or MIAPaCa-2 cells (30). In both models, the neutralization of the C3a produced by the implanted tumors using human anti-C3a antibody results in a delay of tumor growth (Fig. 4A and B). MIAPaCa-2 tumors had a more significant response to anti-C3a antibody, most probably due to lower levels of C3a secretion. Furthermore, in the PANC-1 xenograft model, subsequent inhibition of the C3a receptor with the small-molecule antagonist SB290157 also resulted in a tumor growth delay (Fig. 4C; ref. 31). These data indicate that the inhibition of the C3a/C3aR axis through either the depletion of tumor-secreted C3a or blockade of C3aR leads to significant delay in tumor growth. The fact that these immune-deficient mice only have functional NK cells but not T cells allows us to hypothesize that NK cells might be the effector cells mediating the observed tumor growth delay. Consistent with that hypothesis, analysis of tumors by FACS or immunofluorescence (IF) staining revealed an increase in intratumoral NK cells (CD49b^+^ or NCR1^+^) following C3a depletion compared with vehicle treatment (Fig. 4D and E).

### C3aR Antagonism Delays Tumor Growth in a Pan02 Syngeneic Mouse Model of Pancreatic Cancer

**FIGURE 5:**
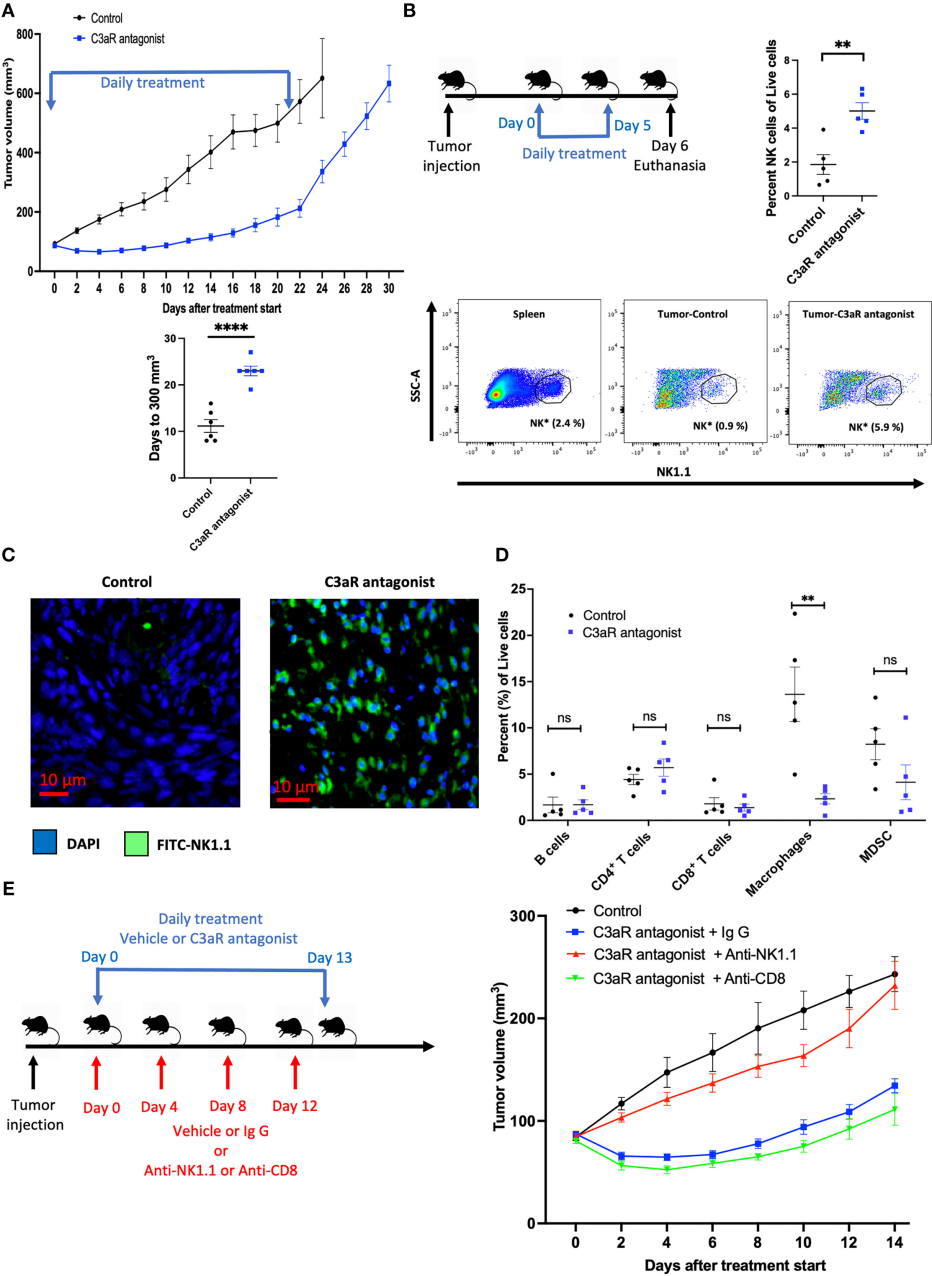
C3a receptor antagonism results in tumor growth delay of Pan02 syngeneic mouse model in C57BL/6 mice and is mediated by increased NK cell infiltration in the TME. **A,** After Pan02 tumor cells implanted subcutaneously in C57BL/6 mice reached a volume of approximately 100 mm^3^, treatment was started with either daily intraperitoneal injection of vehicle (4% DMSO in PBS; control group, *n* = 6) or with daily intraperitoneal injection of the small-molecule C3aR antagonist SB290157 (SB290157 group, 5 mg/kg, *n* = 6) for 22 days and tumor size was monitored during and after treatment until endpoint (tumor ulceration or volume >1,000 mm^3^). The mean time to reaching 300 mm^3^ in the control group was 11.2 days compared with 23 days in the SB902157 treatment group (****, *P* < 0.0001). **B,** Flow cytometry analysis of control versus C3aR antagonist treated Pan02 tumor-bearing mice. Mice were randomized in two treatment groups (control vs. C3aR antagonist, *n* = 5 per group) and were treated daily for six treatments and euthanized the day after the last treatment. C3aR antagonism results in an increased NK cells (NK1.1^+^; as a percentage of live cells) in the TME (**, *P* = 0.0032). Representative gating of NK cells shown (^*^, Proportion of NK cells as a percentage of live cells). **C,** Representative IF staining of control and C3aR antagonist–treated mice (*n* = 3 per treatment group), showing an increase of NK cells (NK1.1-FITC) in the TME of C3aR antagonist-treated mice compared with vehicle-treated mice. **D,** Flow cytometry analysis of control versus C3aR antagonist–treated Pan02 tumor-bearing mice. Except a decrease in macrophages (Gr1^−^CD11b^+^F4/80^+^) in the C3aR antagonist–treated tumors (**, *P* = 0.0055), no statistically significant (ns) difference in B cells (B220^+^), CD4^+^ T cells, CD8^+^ T cells, and MDSCs (Gr1^+^CD11b^+^) is noted between the two treatment groups. **E,** Left: NK and CD8^+^ T cells depletion experiment scheme. After randomization into the treatment arms, NK cells or CD8^+^ T cells depletion was achieved by the administration of 100 μg of antibody (anti-NK1.1, or anti-CD8) intraperitoneal on days 0, 4, 8, and 12. Mouse IgG1 was used as isotype control. Mice undergoing NK and CD8^+^ T cells depletion and receiving IgG1 isotype control antibody were treated with daily intraperitoneal injection of SB290157 (5 mg/kg) from day 0 to day 13. Mice in the control arm were treated with daily vehicle (4% DMSO in PBS). Right: Depletion of NK cells abrogates the effect of the C3aR antagonist, resulting in tumor growth rate similar to the control group, whereas with CD8^+^ T cells depletion, C3aR antagonist retains its effect on tumor growth delay. Statistical significance determined using *t* test.

While the use of xenograft models in T cell–deficient animals allows us to assess the effect of altering the NK cell population, this approach is not optimal for the study of the complex immune interactions in an immunocompetent organism. As such, we sought to evaluate the effect of the C3a/C3aR axis inhibition in a syngeneic mouse model of pancreatic cancer. Pan02 is a murine pancreatic cancer cell line established in C57BL/6 mice (32). The syngeneic model of Pan02 in C57BL/6 mouse is characterized by dense stroma and significant desmoplasia, as demonstrated by H&E and trichrome stains (Supplementary Fig. S3a) and is devoid of NK cells. We initially determined the suitability of Pan02 syngeneic mouse model for this study by first evaluating the secretion of C3a by Pan02 cells and whether NK cells isolated from C57BL/6 mice are cytotoxic to Pan02 cells. Indeed, C3a was detected in the supernatant of Pan02 cells by ELISA (Supplementary Fig. S3b) and using the LDH release assay, we demonstrated that these NK cells possess cytotoxic activity against Pan02 cells at ratio of NK cells to tumor cells ranging from 1.25:1 to 20:1 (Supplementary Fig. S3c). In C57BL/6 mice bearing subcutaneous Pan02 tumors, daily intraperitoneal injection of the C3aR antagonist small molecule SB290157 (5 mg/kg) resulted in significant tumor growth delay. To reach a tumor size of 500 mm^3^, it took on average, 17.8 days for vehicle-treated mice compared with 26.5 days in C3aR antagonist–treated mice (*P* = 0.0019; Fig. 5A).

### The Tumor Growth Delay Effect of the C3aR Antagonist SB290157 in the Pan02 Syngeneic Mouse Model is Mediated by NK Cells

FACS analysis performed on C3aR antagonist–treated mice revealed an increase of NK cells (NK 1.1^+^) in the TME of these tumors compared with those in the vehicle-treated arm (Fig. 5B; Supplementary Fig. S4). This observation was also confirmed by IF (Fig. 5C). We also did not observe a change in cytotoxic CD8^+^ T cells between the two treatment arms (Fig. 5D). Except for a decrease in macrophages (Gr1^−^CD11b^+^F4/80^+^) in the TME following C3aR antagonism, no statistically significant difference was noted with B cells (B220^+^), CD4^+^ T cells and MDSCs (Gr1^+^CD11b^+^; Fig. 5D; Supplementary Fig. S5). The aforementioned changes in the NK cells and macrophages ensuing from the C3aR antagonism were only observed in the TME as no difference was noted in the splenic immune cell populations between the two arms (Supplementary Fig. S6). In addition to showing increased NK cells number with C3aR inhibition using FACS and IF, we also show that NK cell depletion abrogates the effect of the C3aR antagonist on tumor growth delay whereas the depletion of CD8^+^ T cells does not (Supplementary Fig. S7; Fig. 5E, right). Taken together, these data suggest that the tumor growth delay from C3aR inhibition in a syngeneic pancreatic cancer model is due to an increase NK cell infiltration in the TME.

### Radiation is Detrimental to the Cytotoxic Activity and Viability of NK Cells but Enhances the Killing of Malignant Cells by NK Cells

**FIGURE 6:**
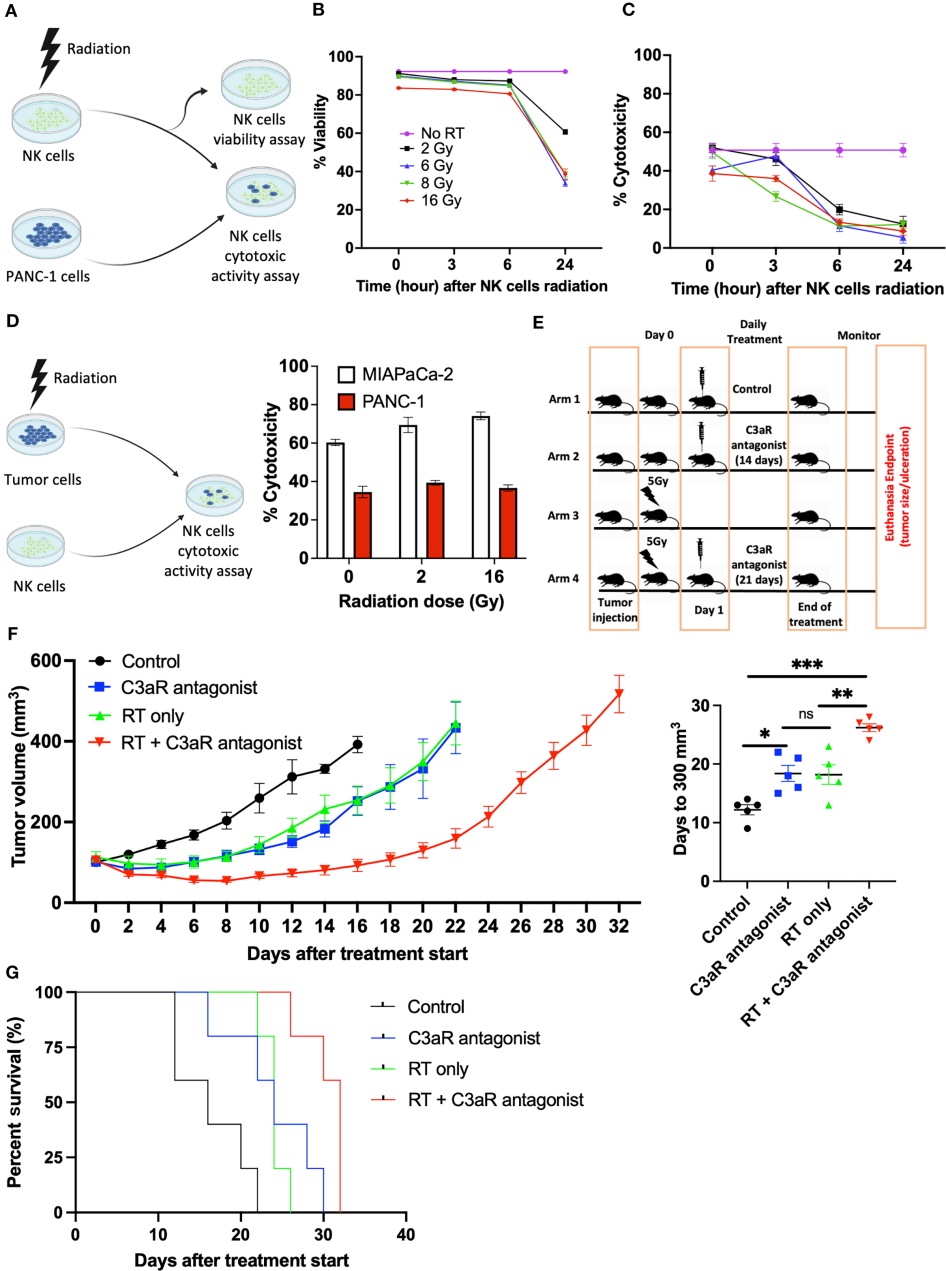
Radiation of NK cells is detrimental to their cytotoxic activity and viability but potentiates the tumor growth delay of C3aR inhibition when delivered before the C3aR antagonist by enhancing NK cell killing of malignant cells. **A,** Experimental design evaluating the impact of radiation on NK cell viability and cytotoxic activity. **B,** The viability of NK cells is significantly decreased 24 hours after various single dose of radiation (*n* = 3, triplicate measurements from two independents experiments). **C,** Radiation of NK cells with various single dose decreases their cytotoxic activity against PANC-1 cells (*n* = 3, triplicate measurements from two independents experiments). The decrease in the cytotoxic activity is observed 3 hours after radiation. Cytotoxic activity measured using the LDH release assay at a ratio of 20:1 (NK:PANC-1). **D,** The killing of both PANC-1 and MIAPaCa-2 cells by NK cells is modestly enhanced by their irradiation prior to NK cell cytotoxic activity assay. Cytotoxic activity measured using the LDH release assay at a ratio of 5:1 (NK:Tumor). **E,** Experimental design in syngeneic mouse model of pancreatic cancer in C57BL/6 mice: After Pan02 tumor subcutaneously implanted in C57BL/6 mice reached approximately 100 mm^3^, the mice were randomized in four treatment arms. Arm 1 (control, intraperitoneal injection of vehicle: 4% DMSO in PBS, *n* = 5), arm 2 (C3aR antagonist, SB290157 only, daily intraperitoneal, 14 days), arm 3 (single radiation dose of 5 Gy, *n* = 5), arm 4 (combination therapy, single fraction of 5 Gy on day 0, followed by daily intraperitoneal injection of SB290157 starting on day 1 for 21 days, *n* = 5). Tumor size was monitored until experimental endpoint (tumor ulceration or volume >1,000 mm^3^). **F,** Left: Tumor growth curve in syngeneic mouse model of pancreatic cancer in C57BL/6 mice with Pan02 cells showing that the combination of C3aR antagonism following radiation results in a significant tumor growth delay compared with each individual treatment. Right: Time (days) after the beginning of treatment to reach 300 mm^3^ was determined; Control arm: 12.2 days; C3aR antagonist (SB290157) only arm: 18.4 days; Radiation only arm: 18.2 days; Combination treatment arm (radiation + C3aR antagonist): 26 days. *, *P* < 0.01; **, *P* < 0.001; ***, *P* < 0.0001; ns, not significant. Statistical significance determined by two-way ANOVA multiple comparisons. **G,** Survival curves of the various treatment arms in the syngeneic mouse model of pancreatic cancer in C57BL/6 mice with Pan02 cells. Median survival: Control (16 days); radiotherapy only (24 days); C3aR antagonist only (24 days); RT + C3aR antagonist (32 days).

While the inhibition of the C3a/C3aR axis results in tumor growth delay, it does not lead to complete tumor regression. This suggests that this approach should be combined with another cancer treatment modality such as radiotherapy to increase the ability to eliminate pancreatic cancer. To address this hypothesis, we sought to investigate the antitumor effect of C3aR antagonist when combined with radiotherapy and the optimal timing of such combination with radiotherapy (e.g., whether to administer the C3aR antagonist before or after radiotherapy). If the C3aR antagonist was administered before radiotherapy, the infiltrated NK cells would be exposed to the subsequent radiotherapy. Thus, it is important to investigate the effect of radiotherapy on the cytotoxic activity and viability of NK cells. These experiments are outlined in Fig. 6A. We found that a single dose of radiotherapy significantly reduced NK cell viability between 6 and 24 hours after radiotherapy treatment (Fig. 6B) and decreased NK cytotoxic activity against malignant cells as early as 3 hours after radiotherapy delivery (Fig. 6C). We next investigated the impact of irradiating both PANC-1 and MIAPaCa-2 cells prior to their exposure to NK cells, which constitutes the scenario in which the C3aR antagonist treatment is initiated after radiotherapy (Fig. 6D, left). A single dose of radiotherapy leads to a modest increase in both PANC-1 and MIAPaCa-2 cells killing by NK cells (Fig. 6D, right). As such, these data suggest that the optimal sequencing of the combination of C3aR antagonist and radiotherapy should be radiotherapy followed by C3aR antagonist treatment to avoid the detrimental effects of radiotherapy on NK cells while harnessing the radiotherapy sensitization of malignant cells to NK cell killing.

### Radiation Potentiates C3aR Antagonism in the Pan02 Syngeneic Mouse Model of Pancreatic Cancer

To evaluate the potentiation of C3aR antagonism by radiotherapy, we compared tumor growth delay ensuing from the combination of a single dose of 5 Gy followed 24 hours later by daily intraperitoneal injection of the C3aR antagonist to treatment arms comprised of radiotherapy only, C3aR antagonist only, and a no-treatment control arm (vehicle only; Fig. 6E). We found that a single dose of 5 Gy has the same effect on tumor growth delay as a 14-day treatment course with the C3aR antagonist alone. However, the combination of both therapies further delays tumor growth compared with each modality alone (Fig. 6F, left). To reach a tumor size of 300 mm^3^, it took an average of 12.2 days for mice in the control arm, compared with 18.4 days for the radiotherapy only or C3aR antagonist only treatment arms, and 26 days for the combination arm (Fig. 6F, right). Furthermore, median survival was 16, 24, 24, and 32 days, respectively, for the control arm, radiotherapy only arm, C3aR antagonist arm and the combination arm, respectively (Fig. 6G). Taken together, these data suggest that although the inhibition of the C3a/C3aR axis represents a promising immunotherapeutic option against pancreatic cancer, its combination with radiotherapy, delivered prior to the C3a/C3aR inhibition holds a greater therapeutic benefit.

## Discussion

Because the discovery of NK cells and understanding of their cytotoxicity against malignant and virus-infected cells, significant efforts have emerged to harness their activity in cancer immunotherapy. Numerous strategies to enhance NK cell anticancer activity are presently being tested in NK cell–based immunotherapy clinical trials against hematologic and solid tumors including pancreatic cancer (33). With growing evidence supporting the finding that intratumoral NK cell density correlates with survival in various cancer types, NK cell–based immunotherapy aimed at enhancing NK cell infiltration in the TME holds a significant therapeutic benefit including in pancreatic cancer (19).

By applying CIBERSORTx analysis to RNA-seq data of patients with pancreatic cancer in TCGA database, we found that similar to other cancer types, high density of NK cells in the TME correlates with improved patient survival. Although the association between intratumoral CD8^+^ T cells and overall survival was previously observed in patients with cutaneous melanoma using CIBERSORT, this current study represents to our knowledge the first use of this analytic method to evaluate the correlation between intratumoral NK cells and overall survival in patients with pancreatic cancer (34). In addition, analysis of our institutional pancreatic cancer database also indicates that in most patients, NK cells are absent from the TME. However, this analysis did not reveal a correlation between patient survival and number of NK cells, which could be attributed to a sampling error due to the very small amount of tissue, an inherent limitation to the use of TMAs. This limitation may have an even greater impact on evaluating markers with potential intratumor spatial heterogeneity (35). Nevertheless, a key point gleaned from this analysis of 192 patients is that the intratumoral density of NK cells is very low in pancreatic cancer. Thus, a better understanding of the mechanism of NK cell exclusion is warranted.

Andoh and colleagues demonstrated that complement C3, precursor of C3a is produced and secreted by pancreatic cancer cells, PANC-1 and MIAPaCa-2 (36). In addition to the known complement activation pathways such as the classical, lectin, and alternative pathways, C3 can also be cleaved into C3a by the enzymatic activity of cathepsins which are upregulated in pancreatic cancer (37, 38). As such, our detection of C3a in the supernatant of the pancreatic cancer cell lines PANC-1 and MIAPaCa-2 is consistent with the aforementioned literature.

Unlike previously reported by Charriaut and colleagues, C3a does not inhibit the cytotoxic activity of NK cells (29). Such discrepancy may reside in the methods of NK cell isolation. Charriaut and colleagues used a Ficoll/isopaque gradient centrifugation to isolate mononuclear cells followed by Percoll density gradients to isolate NK cells resulting in potential contamination (29). In contrast, we applied a negative selection based on antibody targeted and removal of other immune cell types while enriching the NK cell population and yielding highly purified NK cells.

Previous work by Janelle and colleagues demonstrated that the depletion of the complement C3 in melanoma-bearing mice, resulted in an increase infiltration of NK cells within the TME and subsequent tumor growth delay (39). Although, this study provided a novel role of the complement system in the regulation of NK cell function, the key component of the complement system responsible for such regulation was not identified as the depletion of C3 alters the formation of other peptides of the complement system (20). Our previous publication subsequently showed the involvement of the C3a/C3aR axis in the exclusion of NK cells from the TME of breast and colorectal cancer mouse models (21).

In addition to the increase in NK cells in the TME of C3aR antagonist–treated mice, a decrease in macrophages was also noted with C3aR antagonist treatment. Although, we did not evaluate the polarity of these tumor-associated macrophages to determine the relative proportion of M1 (proinflammatory) compared with M2 (anti-inflammatory), the literature suggests that in pancreatic cancer the M2 types predominate in the TME (40). As such, the ensuing decreased in macrophages following C3aR antagonist treatment may be beneficial in ameliorating the immunosuppressive TME of pancreatic cancer.

The observation that C3a/C3aR axis inhibition alone does not result in complete tumor regression of pancreatic cancer is consistent with the literature suggesting that although NK cells are capable of complete eradication of hematopoietic malignancies, they are unable to eradicate solid tumors including in preclinical models of pancreatic cancer (41–44). Thus, for NK cell–based immunotherapy to be successful against solid tumors including pancreatic cancer, a combination with other cancer treatment modality will be needed.

Because of the ensuing pancytopenia from systemic chemotherapy including lymphopenia, we elected to explore the combination of radiotherapy with C3a/C3aR axis inhibition to enhance NK cell–based immunotherapy against pancreatic cancer. Radiotherapy delivers its therapeutic effect locally in the radiation field, thus avoiding lymphopenia and also possesses immunostimulatory effects. These effects include immunogenic cell death characterized by the translocation of calreticulin to the cell surface, the release of ATP and high mobility group box 1 (HMGB 1) protein in the extracellular milieu, which promotes the influx of immune cells in the TME (45, 46). The activation of DNA damage checkpoint pathway initiated by ataxia telangiectasia mutated (ATM) or ataxia telangiectasia mutated and rad3-related (ATR) kinases following radiotherapy leads to the upregulation of the ligands of the NK cell–activating receptor NKG2D on malignant cells thus enhancing NK cell cytotoxic activity (47). Radiotherapy provides additional immunostimulatory stimuli by increasing the expression of MHC-I molecules on the surface and the intracellular peptides which facilitate the immune system recognition (48).

On the other hand, radiotherapy also possesses immunosuppressive effects, some of which are mediated by complement C3a. Radiotherapy induces the local production of C3a which is further enhanced by radiotherapy-mediated cell death (49, 50). Thus, the combination of radiotherapy and C3a/C3aR axis inhibition may enable the harnessing of the immunostimulatory effects of radiotherapy while minimizing the C3a-mediated immunosuppressive effects. However, a critical question which has plagued the combination of radiotherapy and other immunotherapy agents is the optimal sequence of the combination; radiotherapy before or after C3a/C3aR axis inhibition. Although we showed that when using a single fraction dose, radiotherapy treatment prior to C3aR inhibition may be the optimal timing for the combination therapy, resulting in a potentiation of C3aR antagonism, the question remains when multi-fraction regimens of radiotherapy are desired. A single fraction of 5 Gy may not be the optimal radiation dose and fractionation as clinically used doses and fractionation of stereotactic ablative radiotherapy for pancreatic cancer include 30–45 Gy in 3 fractions or 25–45 Gy in 5 fractions (51). Thus, a higher dose of radiation per fraction for a higher total dose in combination with C3aR antagonism may result in further tumor growth delay or complete tumor regression.

In summary, we demonstrate that higher density of NK cells in the TME of patients with pancreatic cancer correlates with improved survival. However, the TME of most patients with pancreatic cancer is devoid of NK cells. We subsequently show that complement C3a is involved in the exclusion of NK cells from the TME and the inhibition of the C3a/C3aR axis in a syngeneic mouse model of pancreatic cancer restores NK cell infiltration resulting in tumor growth delay. However, complete tumor response to C3a/C3aR axis inhibition by monotherapy is not achievable. We show that radiotherapy potentiates the effect of the C3aR inhibition leading to a more significant tumor growth delay than C3aR inhibition monotherapy. Taken together, these data suggest that the inhibition of C3aR should be tested as a new immunotherapeutic approach for NK cell–based immunotherapy against pancreatic cancer, but for optimal results, a combination with radiotherapy is needed.

## Supplementary Material

Supplementary Figures S1-S7 Supplementary Figure S1: Proportion of total NK cell in the tumor microenvironment of 138 pancreatic cancer patients based on the CIBERSORTx analysis of TCGA. Supplementary Figure S2: Immunohistochemical staining of NK cells (Red circle) using NKp46. Supplementary Figure S3: Pan02 tumor in C57BL/6 mice as a syngeneic mouse model. Supplementary Figure S4: Representative flow cytometry gating strategy for NK cells (a) and percent of NK cells relative to immune cells in the C3aR antagonist-treated and vehicle-treated tumors (b). Supplementary Figure S5: Flow cytometry gating strategy for B cells (B220+), CD4+ T cells, CD8+ T cells, macrophages (Gr1-CD11b+F4/80+) and myeloid derived suppressor cells (MDSC) (Gr1+CD11b+) in the spleen, vehicle-treated (control) tumor and C3aR antagonist-treated tumor. Supplementary Figure S6: C3aR antagonism does not impact immune cell infiltration in the spleen of vehicle-treated and C3aR antagonist-treated mice. Supplementary Figure S7: Depletion of NK cells and CD8+ T cells and confirmatory analysis of depletion and survival of Pan02 tumor-bearing mice.

Supplementary Table S1 Proportion of various immune cell type in TME of pancreatic cancer (TGCA).

## References

[1] Siegel RL , Miller KD, Fuchs HE, Jemal A. Cancer statistics, 2021. CA Cancer J Clin 2021;71:7–33. 33433946 10.3322/caac.21654

[2] Rahib L , Smith BD, Aizenberg R, Rosenzweig AB, Fleshman JM, Matrisian LM. Projecting cancer incidence and deaths to 2030: the unexpected burden of thyroid, liver, and pancreas cancers in the United States. Cancer Res 2014;74:2913–21. 24840647 10.1158/0008-5472.CAN-14-0155

[3] Sung H , Ferlay J, Siegel RL, Laversanne M, Soerjomataram I, Jemal A, . Global Cancer Statistics 2020: GLOBOCAN estimates of incidence and mortality worldwide for 36 cancers in 185 countries. CA Cancer J Clin 2021;71:209–49. 33538338 10.3322/caac.21660

[4] Pourshams A , Sepanlou SG, Ikuta KS, Bisignano C, Safiri S, Roshandel G, . The global, regional, and national burden of pancreatic cancer and its attributable risk factors in 195 countries and territories, 1990–2017: a systematic analysis for the Global Burden of Disease Study 2017. Lancet Gastroenterol Hepatol 2019;4:934–47. 31648972 10.1016/S2468-1253(19)30347-4 PMC7026711

[5] Huang J , Lok V, Ngai CH, Zhang L, Yuan J, Lao XQ, . Worldwide burden of, risk factors for, and trends in pancreatic cancer. Gastroenterology 2021;160:744–54. 33058868 10.1053/j.gastro.2020.10.007

[6] Robert C . A decade of immune-checkpoint inhibitors in cancer therapy. Nat Commun 2020;11:3801. 32732879 10.1038/s41467-020-17670-y PMC7393098

[7] Yu JX , Hubbard-Lucey VM, Tang J. Immuno-oncology drug development goes global. Nat Rev Drug Discov 2019;18:899–900. 31780841 10.1038/d41573-019-00167-9

[8] Andersson R , Pereira C-F, Bauden M, Ansari D. Is immunotherapy the holy grail for pancreatic cancer? Immunotherapy 2019;11:1435–8. 31747808 10.2217/imt-2019-0164

[9] Morrison AH , Byrne KT, Vonderheide RH. Immunotherapy and prevention of pancreatic cancer. Trends Cancer 2018;4:418–28. 29860986 10.1016/j.trecan.2018.04.001 PMC6028935

[10] Schizas D , Charalampakis N, Kole C, Economopoulou P, Koustas E, Gkotsis E, . I mmunotherapy for pancreatic cancer: a 2020 update. Cancer Treat Rev 2020;86:102016. 32247999 10.1016/j.ctrv.2020.102016

[11] Zhang C , Hu Y, Shi C. Targeting natural killer cells for tumor immunotherapy. Front Immunol 2020;11:60. 32140153 10.3389/fimmu.2020.00060 PMC7042203

[12] Creelan BC , Antonia SJ. The NKG2A immune checkpoint - a new direction in cancer immunotherapy. Nat Rev Clin Oncol 2019;16:277–8. 30824813 10.1038/s41571-019-0182-8

[13] Coca S , Perez-Piqueras J, Martinez D, Colmenarejo A, Saez MA, Vallejo C, . The prognostic significance of intratumoral natural killer cells in patients with colorectal carcinoma. Cancer 1997;79:2320–8. 9191519 10.1002/(sici)1097-0142(19970615)79:12<2320::aid-cncr5>3.0.co;2-p

[14] Ishigami S , Natsugoe S, Tokuda K, Nakajo A, Che X, Iwashige H, . Prognostic value of intratumoral natural killer cells in gastric carcinoma. Cancer 2000;88:577–83. 10649250

[15] Takanami I , Takeuchi K, Giga M. The prognostic value of natural killer cell infiltration in resected pulmonary adenocarcinoma. J Thorac Cardiovasc Surg 2001;121:1058–63. 11385371 10.1067/mtc.2001.113026

[16] Villegas FR , Coca S, Villarrubia VG, Jiménez R, Chillón María, Jareño J, . Prognostic significance of tumor infiltrating natural killer cells subset CD57 in patients with squamous cell lung cancer. Lung Cancer 2002;35:23–8. 11750709 10.1016/s0169-5002(01)00292-6

[17] Cózar JM , Canton J, Tallada M, Concha A, Cabrera T, Garrido F, ., Analysis of NK cells and chemokine receptors in tumor infiltrating CD4 T lymphocytes in human renal carcinomas. Cancer Immunol Immunother 2005;54:858–66. 15887015 10.1007/s00262-004-0646-1 PMC11032824

[18] Hsia JY , Chen JT, Chen CY, Hsu CP, Miaw J, Huang YS, . Prognostic significance of intratumoral natural killer cells in primary resected esophageal squamous cell carcinoma. Chang Gung Med J 2005;28:335–40. 16086548

[19] Nersesian S , Schwartz SL, Grantham SR, Maclean LK, Lee SN, Pugh-Toole M, . NK cell infiltration is associated with improved overall survival in solid cancers: a systematic review and meta-analysis. Transl Oncol 2021;14:100930. 33186888 10.1016/j.tranon.2020.100930 PMC7670197

[20] Ricklin D , Hajishengallis G, Yang K, Lambris JD. Complement: a key system for immune surveillance and homeostasis. Nat Immunol 2010;11:785–97. 20720586 10.1038/ni.1923 PMC2924908

[21] Nandagopal S , Li CG, Xu Y, Sodji QH, Graves EE, Giaccia AJ. C3aR signaling inhibits NK cell infiltration into the tumor microenvironment in mouse models. Cancer Immunol Res 2022;10:245–58. 34819308 10.1158/2326-6066.CIR-21-0435 PMC9351714

[22] Newman AM , Steen CB, Liu CL, Gentles AJ, Chaudhuri AA, Scherer F, . Determining cell type abundance and expression from bulk tissues with digital cytometry. Nat Biotechnol 2019;37:773–82. 31061481 10.1038/s41587-019-0114-2 PMC6610714

[23] Chiou S-H , Dorsch M, Kusch E, Naranjo S, Kozak MM, Koong AC, . Hmga2 is dispensable for pancreatic cancer development, metastasis, and therapy resistance. Sci Rep 2018;8:14008. 30228296 10.1038/s41598-018-32159-x PMC6143627

[24] Hernández JM , Bui MHT, Han K-r, Mukouyama H, Freitas DG, Nguyen D, . Novel kidney cancer immunotherapy based on the granulocyte-macrophage colony-stimulating factor and carbonic anhydrase IX fusion gene. Clin Cancer Res 2003;9:1906–16. 12738749

[25] Radenkovic M , Uvebrant K, Skog O, Sarmiento L, Avartsson J, Storm P, . Characterization of resident lymphocytes in human pancreatic islets. Clin Exp Immunol 2017;187:418–27. 27783386 10.1111/cei.12892 PMC5290249

[26] Muntasell A , Rojo F, Servitja S, Rubio-Perez C, Cabo M, Tamborero D, . NK cell infiltrates and HLA class I expression in primary HER2(+) breast cancer predict and uncouple pathological response and disease-free survival. Clin Cancer Res 2019;25:1535–45. 30523021 10.1158/1078-0432.CCR-18-2365

[27] Taghavi N , Bagheri S, Akbarzadeh A. Prognostic implication of CD57, CD16, and TGF-β expression in oral squamous cell carcinoma. J Oral Pathol Med 2016;45:58–62. 25808210 10.1111/jop.12320

[28] Van Acker HH , Capsomidis A, Smits EL, Van Tendeloo VF. CD56 in the immune system: more than a marker for cytotoxicity? Front Immunol 2017;8:892. 28791027 10.3389/fimmu.2017.00892 PMC5522883

[29] Charriaut C , Senik A, Kolb JP, Barel M, Frade R. Inhibition of *in vitro* natural killer activity by the third component of complement: role for the C3a fragment. Proc Natl Acad Sci U S A 1982;79:6003–7. 6985269 10.1073/pnas.79.19.6003 PMC347040

[30] Shultz LD , Ishikawa F, Greiner DL. Humanized mice in translational biomedical research. Nat Rev Immunol 2007;7:118–30. 17259968 10.1038/nri2017

[31] Ames RS , Lee D, Foley JJ, Jurewicz AJ, Tornetta MA, Bautsch W, . Identification of a selective nonpeptide antagonist of the anaphylatoxin C3a receptor that demonstrates antiinflammatory activity in animal models. J Immunol 2001;166:6341–8. 11342658 10.4049/jimmunol.166.10.6341

[32] Corbett TH , Roberts BJ, Leopold WR, Peckham JC, Wilkoff LJ, Griswold DP, . Induction and chemotherapeutic response of two transplantable ductal adenocarcinomas of the pancreas in C57BL/6 mice. Cancer Res 1984;44:717–26. 6692374

[33] Liu S , Galat V, Galat4 Y, Lee YKA, Wainwright D, Wu J. NK cell-based cancer immunotherapy: from basic biology to clinical development. J Hematol Oncol 2021;14:7. 33407739 10.1186/s13045-020-01014-w PMC7788999

[34] Chen B , Khodadoust MS, Liu CL, Newman AM, Alizadeh AA. Profiling tumor infiltrating immune cells with CIBERSORT. Methods Mol Biol 2018;1711:243–59. 29344893 10.1007/978-1-4939-7493-1_12 PMC5895181

[35] Lee ATJ , Chew W, Wilding CP, Guljar N, Smith MJ, Strauss DC, . The adequacy of tissue microarrays in the assessment of inter- and intra-tumoural heterogeneity of infiltrating lymphocyte burden in leiomyosarcoma. Sci Rep 2019;9:14602. 31601875 10.1038/s41598-019-50888-5 PMC6787212

[36] Andoh A , Fujiyama Y, Sumiyoshi K, Bamba T. Local secretion of complement C3 in the exocrine pancreas: ductal epithelial cells as a possible biosynthetic site. Gastroenterology 1996;110:1919–25. 8964419 10.1053/gast.1996.v110.pm8964419

[37] Keliher EJ , Reiner T, Earley S, Klubnick J, Tassa C, Lee AJ, . Targeting cathepsin E in pancreatic cancer by a small molecule allows *in vivo* detection. Neoplasia 2013;15:684–93. 23814481 10.1593/neo.13276 PMC3689232

[38] Jakoš T , Pišlar A, Jewett A, Kos J. Cysteine cathepsins in tumor-associated immune cells. Front Immunol 2019;10:2037. 31555270 10.3389/fimmu.2019.02037 PMC6724555

[39] Janelle V , Langlois M-P, Tarrab E, Lapierre P, Poliquin L, Lamarre A. Transient complement inhibition promotes a tumor-specific immune response through the implication of natural killer cells. Cancer Immunol Res 2014;2:200–6. 24778316 10.1158/2326-6066.CIR-13-0173

[40] Yang S , Liu Q, Liao Q. Tumor-associated macrophages in pancreatic ductal adenocarcinoma: origin, polarization, function, and reprogramming. Front Cell Dev Biol 2021;8:607209. 33505964 10.3389/fcell.2020.607209 PMC7829544

[41] Klingemann HG . Cellular therapy of cancer with natural killer cells—where do we stand? Cytotherapy 2013;15:1185–94. 23768925 10.1016/j.jcyt.2013.03.011

[42] Ames E , Murphy WJ. Advantages and clinical applications of natural killer cells in cancer immunotherapy. Cancer Immunol Immunother 2014;63:21–8. 23989217 10.1007/s00262-013-1469-8 PMC3880590

[43] Ames E , Canter RJ, Grossenbacher SK, Mac S, Chen M, Smith RC, . NK cells preferentially target tumor cells with a cancer stem cell phenotype. J Immunol 2015;195:4010–9. 26363055 10.4049/jimmunol.1500447 PMC4781667

[44] Stojanovic A , Cerwenka A. Natural killer cells and solid tumors. J Innate Immun 2011;3:355–64. 21502747 10.1159/000325465

[45] Golden EB , Apetoh L. Radiotherapy and immunogenic cell death. Semin Radiat Oncol 2015;25:11–7. 25481261 10.1016/j.semradonc.2014.07.005

[46] Golden E , Pellicciotta I, Demaria S, Barcellos-Hoff MH, Formenti S. The convergence of radiation and immunogenic cell death signaling pathways. Front Oncol 2012;2:88. 22891162 10.3389/fonc.2012.00088 PMC3413017

[47] Gasser S , Orsulic S, Brown EJ, Raulet DH. The DNA damage pathway regulates innate immune system ligands of the NKG2D receptor. Nature 2005;436:1186–90. 15995699 10.1038/nature03884 PMC1352168

[48] Reits EA , Hodge JW, Herberts CA, Groothuis TA, Chakraborty M, K. Wansley E, . Radiation modulates the peptide repertoire, enhances MHC class I expression, and induces successful antitumor immunotherapy. J Exp Med 2006;203:1259–71. 16636135 10.1084/jem.20052494 PMC3212727

[49] Surace L , Lysenko V, Fontana Andrea O, Cecconi V, Janssen H, Bicvic A, . Complement is a central mediator of radiotherapy-induced tumor-specific immunity and clinical response. Immunity 2015;42:767–77. 25888260 10.1016/j.immuni.2015.03.009

[50] Elvington M , Scheiber M, Yang X, Lyons K, Jacqmin D, Wadsworth C, . Complement-dependent modulation of antitumor immunity following radiation therapy. Cell Rep 2014;8:818–30. 25066124 10.1016/j.celrep.2014.06.051 PMC4137409

[51] Tempero MA , Malafa MP, Al-Hawary M, Behrman SW, Benson AB, Cardin DB, . Pancreatic adenocarcinoma, version 2.2021, NCCN clinical practice guidelines in oncology. J Natl Compr Canc Netw 2021;19:439–57. 33845462 10.6004/jnccn.2021.0017

