# Supplementary material for: The Combination of Radiotherapy and Complement C3a Inhibition Potentiates Natural Killer cell Functions Against Pancreatic Cancer
Source: Cancer Res Commun. 2022 Jul 27;2(7):725–38. doi: 10.1158/2767-9764.CRC-22-0069 (PMC9354534; doi:10.1158/2767-9764.CRC-22-0069)
Supplement: Supplementary Figures S1-S7 — Supplementary Figure S1: Proportion of total NK cell in the tumor microenvironment of 138 pancreatic cancer patients based on the CIBERSORTx analysis of TCGA. Supplementary Figure S2: Immunohistochemical staining of NK cells (Red circle) using NKp46. Supplementary Figure S3: Pan02 tumor in C57BL/6 mice as a syngeneic mouse model. Supplementary Figure S4: Representative flow cytometry gating strategy for NK cells (a) and percent of NK cells relative to immune cells in the C3aR antagonist-treated and vehicle-treated tumors (b). Supplementary Figure S5: Flow cytometry gating strategy for B cells (B220+), CD4+ T cells, CD8+ T cells, macrophages (Gr1-CD11b+F4/80+) and myeloid derived suppressor cells (MDSC) (Gr1+CD11b+) in the spleen, vehicle-treated (control) tumor and C3aR antagonist-treated tumor. Supplementary Figure S6: C3aR antagonism does not impact immune cell infiltration in the spleen of vehicle-treated and C3aR antagonist-treated mice. Supplementary Figure S7: Depletion of NK cells and CD8+ T cells and confirmatory analysis of depletion and survival of Pan02 tumor-bearing mice. [file crc-22-0069-s01.pdf]

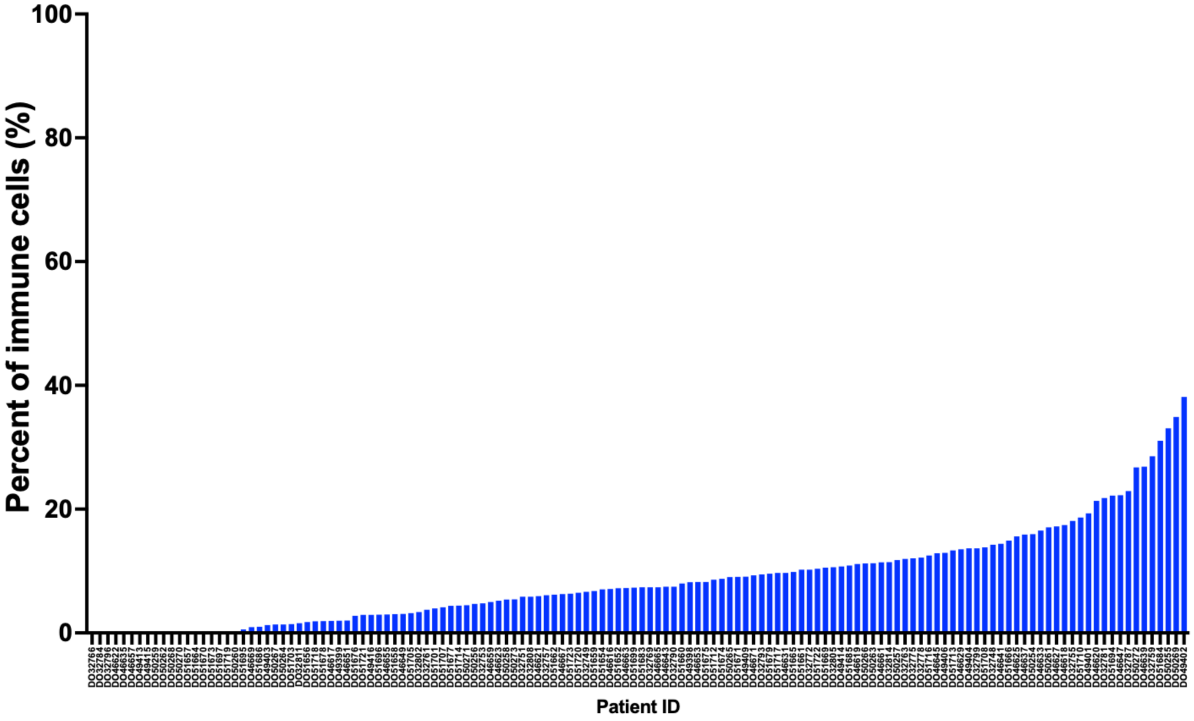

Supplementary Figure S1: Proportion of total NK cell in the tumor microenvironment of 138 pancreatic cancer patients based on the CIBERSORTx analysis of TCGA. Each column represents a pancreatic cancer patient. Total NK cell for each patient was obtained by combining activated and resting NK cells.

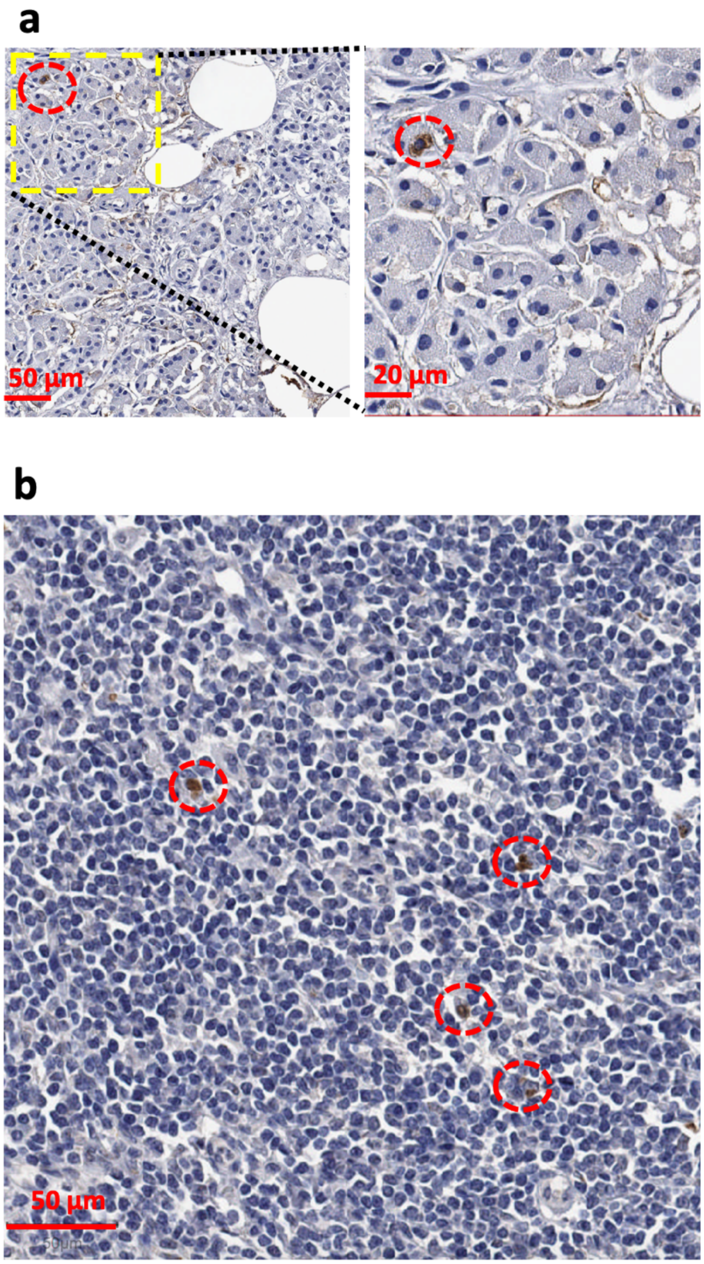

Supplementary Figure S2: Immunohistochemical staining of NK cells (Red circle) using NKp46.

a) Sample positive staining of pancreatic tumor. Left panel: 20X magnification; Right panel: 50X magnification. **b)** Positive staining of NK cells in a human tonsil.

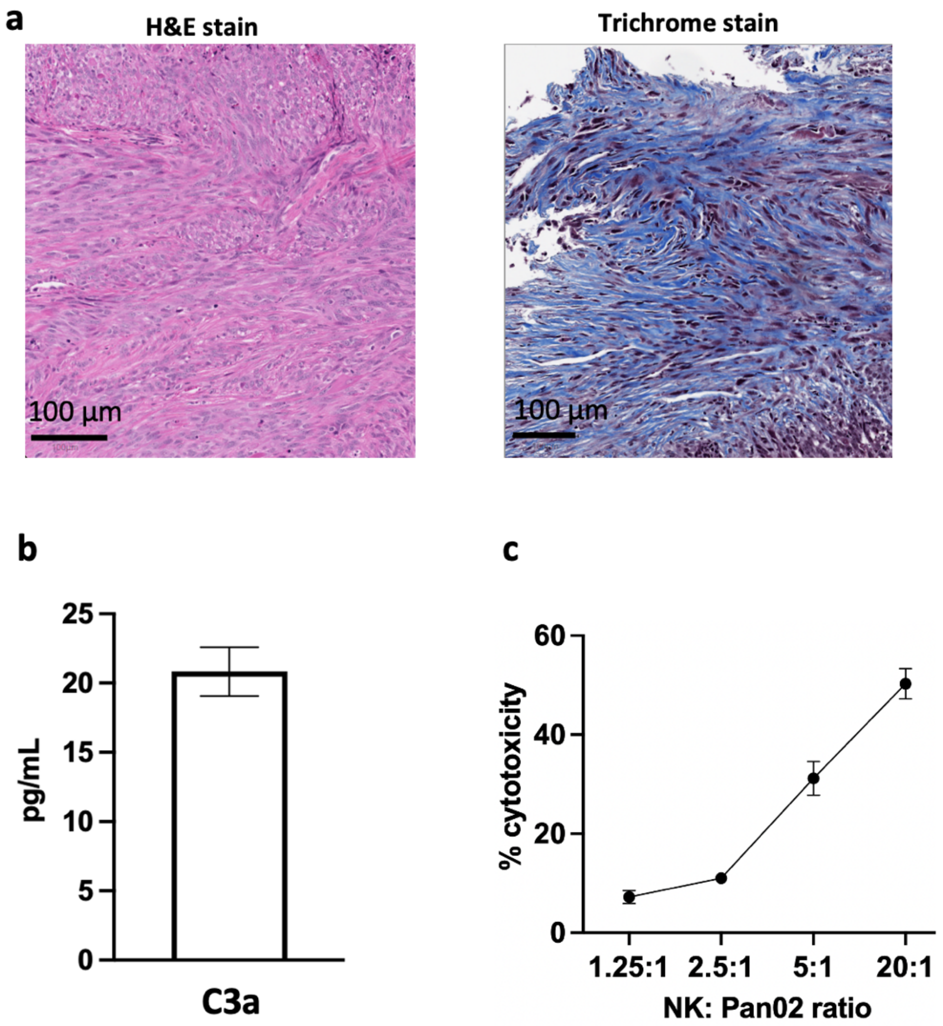

Supplementary Figure S3: Pan02 tumor in C57BL/6 mice as a syngeneic mouse model. (a) Pan02 tumor in C57BL/6 displays significant desmoplasia. Tumors were stained with H&E and trichrome stains (Representative image shown; Magnification: 20X). (**b**) Pan02 cells secrete C3a which was measured by ELISA in the supernatant. No C3a was detected in the growth media (n=3, triplicate measurements from 2 independents experiments). **(c)** NK cells isolated from the spleen of C57BL/6 mice possess cytotoxic activity against Pan02 cells. Cytotoxic activity measured using the LDH release assay at different ratio of NK cells and Pan02 cells (NK:Pan02). (n=3, triplicate measurements from 2 independents experiments)

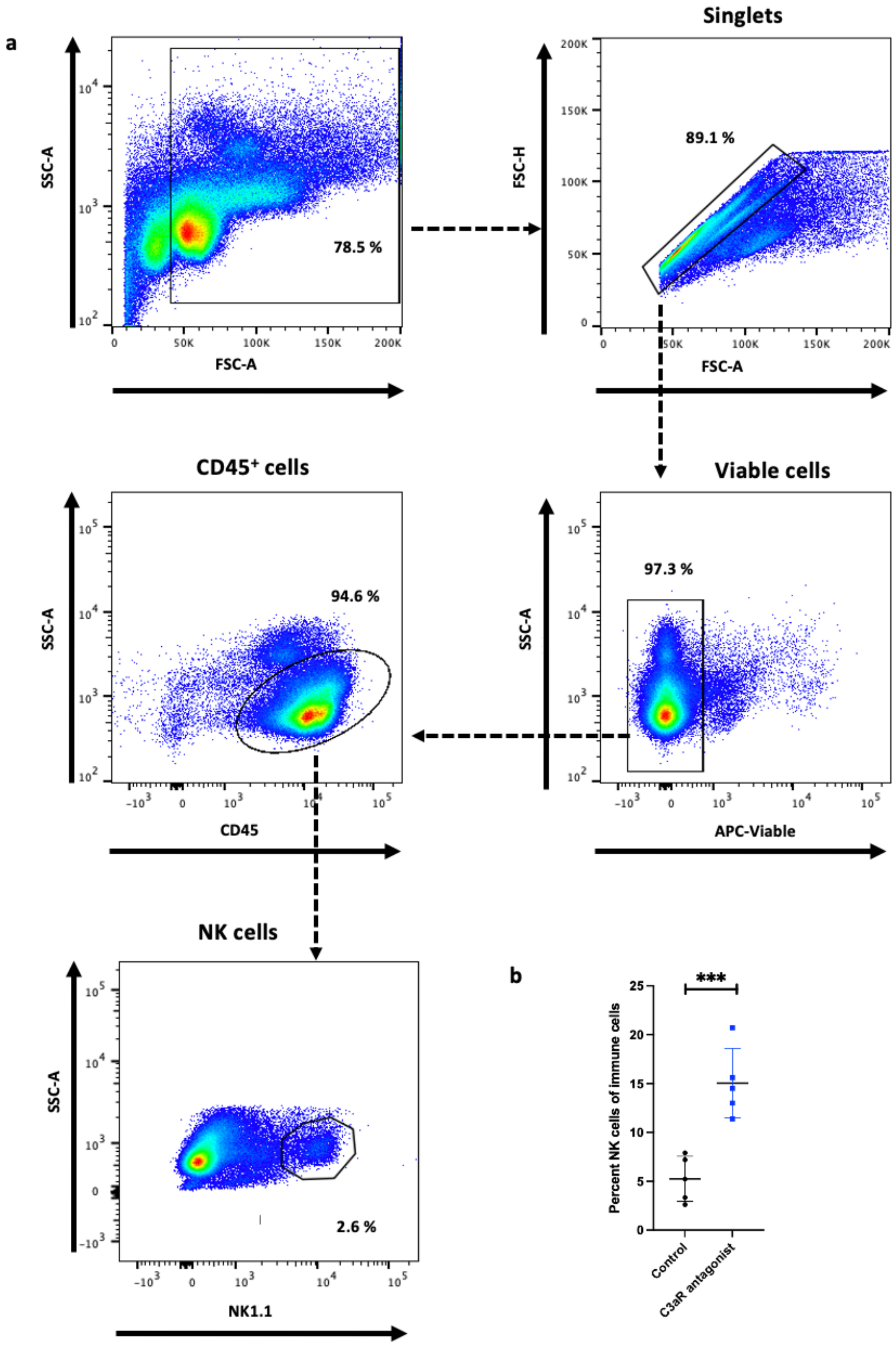
a
SSC-A
FSC-A
78.5 %
Singlets
FSC-H
89.1 %
CD45⁺ cells
CD45
94.6 %
Viable cells
APC-Viable
97.3 %
NK cells
NK1.1
2.6 %
b
Percent NK cells of immune cells
***
Control
C3aR antagonist

Supplementary Figure S4: Representative flow cytometry gating strategy for NK cells (a) and percent of NK cells relative to immune cells in the C3aR antagonist-treated and vehicle-treated tumors (b). The gating strategies shown was applied to a sample (spleen) to identify single cells, viable cells, $CD45^+$ cells and finally NK cells. *** p=0.0008

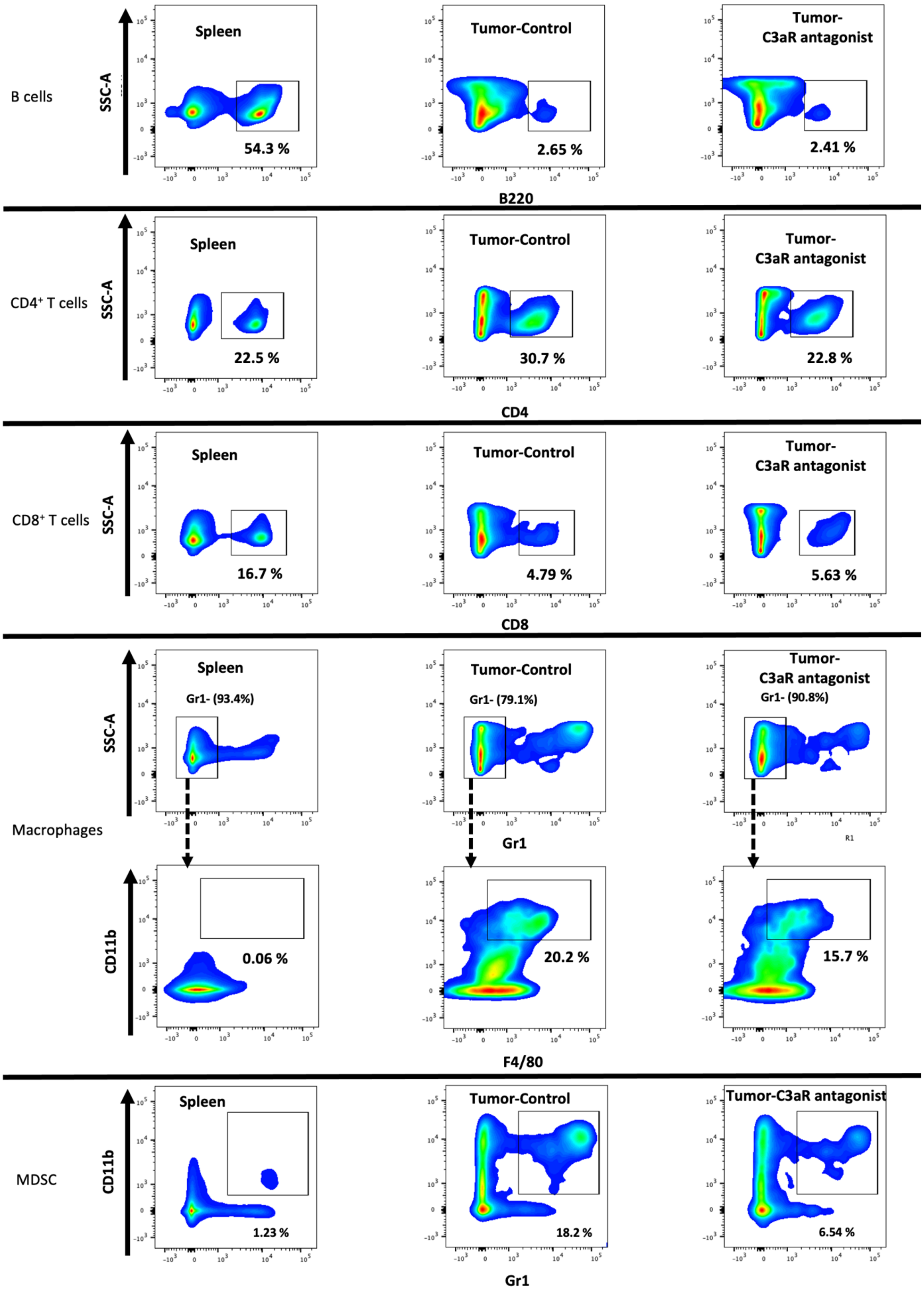
B cells
SSC-A
Spleen
54.3 %
Tumor-Control
2.65 %
Tumor-
C3aR antagonist
2.41 %
B220
CD4⁺ T cells
SSC-A
Spleen
22.5 %
Tumor-Control
30.7 %
Tumor-
C3aR antagonist
22.8 %
CD4
CD8⁺ T cells
SSC-A
Spleen
16.7 %
Tumor-Control
4.79 %
Tumor-
C3aR antagonist
5.63 %
CD8
Macrophages
SSC-A
Spleen
Gr1- (93.4%)
Tumor-Control
Gr1- (79.1%)
Tumor-
C3aR antagonist
Gr1- (90.8%)
Gr1
CD11b
0.06 %
20.2 %
15.7 %
F4/80
MDSC
CD11b
Spleen
1.23 %
Tumor-Control
18.2 %
Tumor-C3aR antagonist
6.54 %
Gr1

Supplementary Figure S5: Flow cytometry gating strategy for B cells (B220^{+}), CD4^{+} T cells, CD8^{+} T cells, macrophages (Gr1^{-}CD11b^{+}F4/80^{+}) and myeloid derived suppressor cells (MDSC) (Gr1^{+}CD11b^{+}) in the spleen, vehicle-treated (control) tumor and C3aR antagonist-treated tumor. Similar gating strategy as shown in supplementary figure S4 was applied to each sample to identify single cells, viable cells and CD45^{+} cells. The gating strategies shown above were applied to the CD45^{+} population.

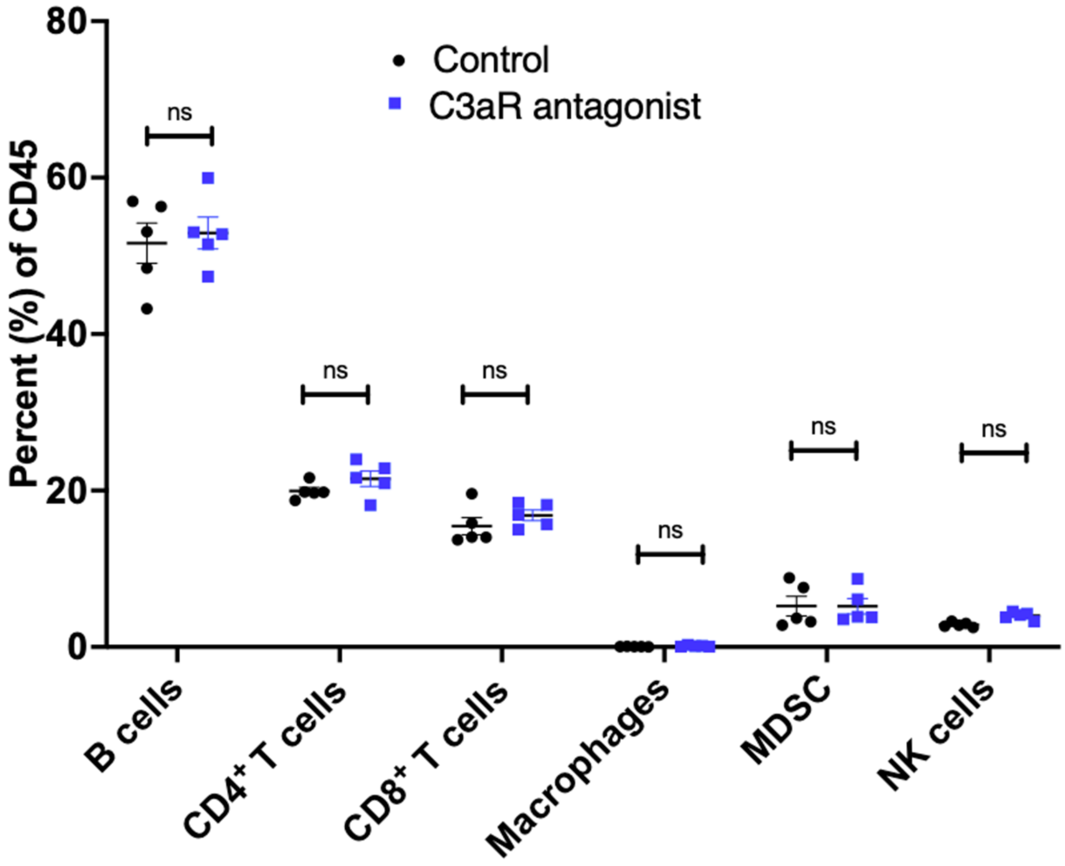

Supplementary Figure S6: C3aR antagonism does not impact immune cell infiltration in the spleen of vehicle-treated and C3aR antagonist-treated mice. Flow cytometry analysis of immune cells isolated from murine spleen in the control vs C3aR antagonist treated mice. Pan02 tumor-bearing mice were randomized in 2 treatment group (control vs C3aR antagonist, n = 5 per group) and were treated daily for 6 treatments and euthanized the following day after the last treatment. The proportion of various immune cells including B cells ($B220^{+}$), $CD4^{+}$ T cells, $CD8^{+}$ T cells, NK cells ($NK1.1^{+}$), macrophages ($Gr1^{-}CD11b^{+}F4/80^{+}$) and myeloid derived suppressor cells (MDSC) ($Gr1^{+}CD11b^{+}$) were analyzed. No difference was observed in the proportion of the aforementioned immune cells in the spleen of C3aR antagonist-treated mice compared to the vehicle- treated mice. n.s: not significant. Statistical significance determined using *t*-test.

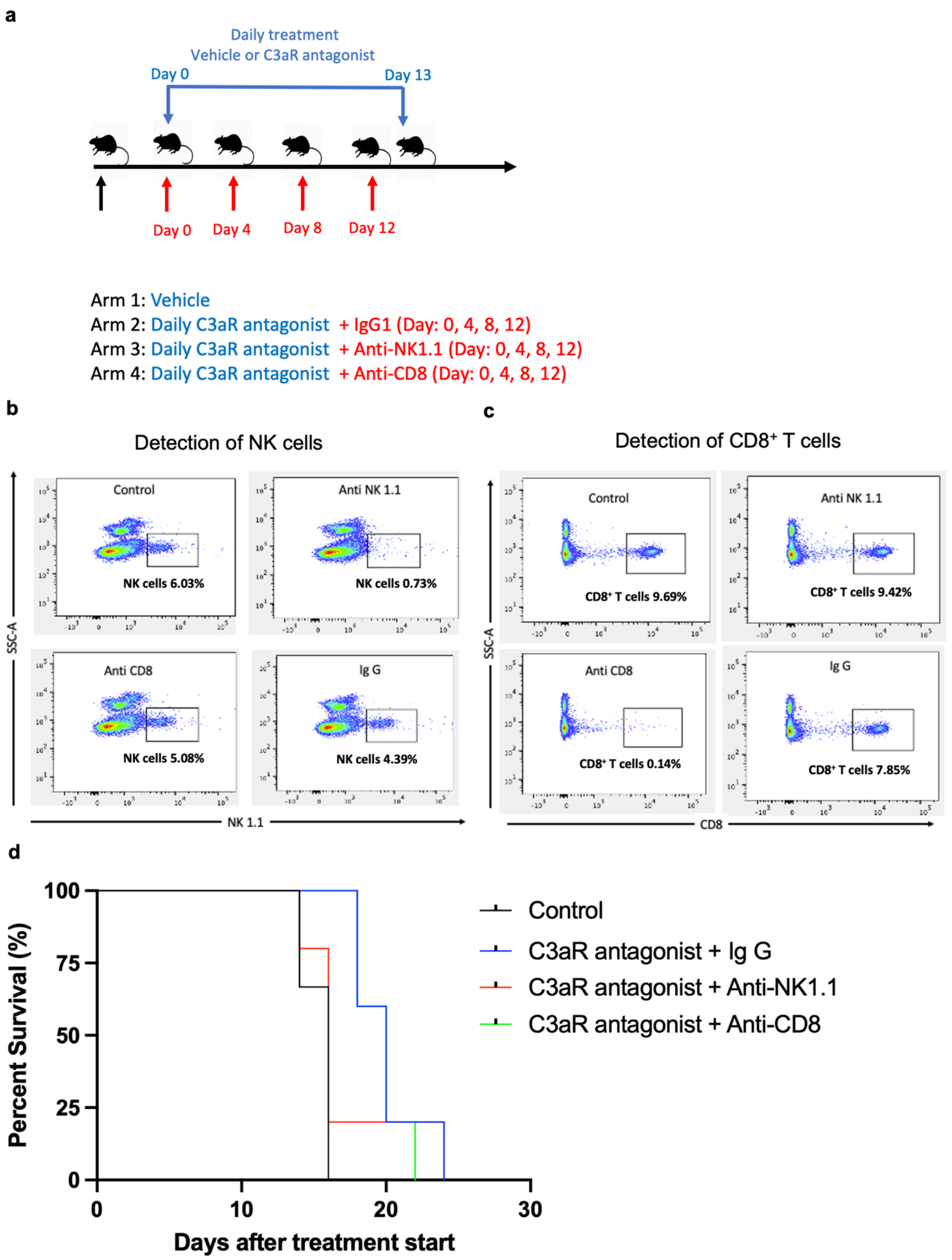
a
Daily treatment
Vehicle or C3aR antagonist
Day 0
Day 13
Day 0
Day 4
Day 8
Day 12
Arm 1: Vehicle
Arm 2: Daily C3aR antagonist + IgG1 (Day: 0, 4, 8, 12)
Arm 3: Daily C3aR antagonist + Anti-NK1.1 (Day: 0, 4, 8, 12)
Arm 4: Daily C3aR antagonist + Anti-CD8 (Day: 0, 4, 8, 12)
b
Detection of NK cells
Control
NK cells 6.03%
Anti NK 1.1
NK cells 0.73%
Anti CD8
NK cells 5.08%
Ig G
NK cells 4.39%
SSC-A
NK 1.1
c
Detection of CD8⁺ T cells
Control
CD8⁺ T cells 9.69%
Anti NK 1.1
CD8⁺ T cells 9.42%
Anti CD8
CD8⁺ T cells 0.14%
Ig G
CD8⁺ T cells 7.85%
SSC-A
CD8
d
Percent Survival (%)
100
75
50
25
0
0
10
20
30
Days after treatment start
Control
C3aR antagonist + Ig G
C3aR antagonist + Anti-NK1.1
C3aR antagonist + Anti-CD8

Supplementary Figure S7: Depletion of NK cells and CD8$^+$ T cells and confirmatory analysis of depletion and survival of Pan02 tumor-bearing mice. The depletion of NK cells or CD8$^+$ T cells was achieved by the administration of 100 µg of antibody (Anti-NK1.1, or Anti-CD8) IP on day 0, 4, 8 and 12. Mouse IgG1 was used as isotype control. **(a)** Schematic of depletion experiment. **(b)** Detection of NK cells by flow cytometry analysis of the peripheral blood of mice in each of the 4 treatment arms (n = 5 per arm) confirming the successful depletion of NK cells. **(c)** Detection of CD8$^+$ T cells by flow cytometry analysis of the peripheral blood of mice in each of the 4 treatment arms (n = 5 per arm) confirming the successful depletion of CD8$^+$ T cells. Percentage of NK or CD8$^+$ T cells in peripheral blood after depletion is shown. **(d)** Survival curves of the various treatment arms. The mice were euthanized due to skin ulceration.
